# MSC Transplantation Improves Lacrimal Gland Regeneration after Surgically Induced Dry Eye Disease in Mice

**DOI:** 10.1038/s41598-019-54840-5

**Published:** 2019-12-04

**Authors:** Jana Dietrich, Lolita Ott, Mathias Roth, Joana Witt, Gerd Geerling, Sonja Mertsch, Stefan Schrader

**Affiliations:** 1 Laboratory of Experimental Ophthalmology, Department of Ophthalmology, Pius-Hospital, Carl-von-Ossietzky University, 26121 Oldenburg, Germany; 2 Laboratory of Experimental Ophthalmology, Department of Ophthalmology, University Hospital Duesseldorf, 40225 Duesseldorf, Germany

**Keywords:** Mesenchymal stem cells, Regeneration

## Abstract

Dry eye disease (DED) is a multifactorial disease characterized by a disrupted tear film homeostasis and inflammation leading to visual impairments and pain in patients. Aqueous-deficient dry eye (ADDE) causes the most severe progressions and depends mainly on the loss of functional lacrimal gland (LG) tissue. Despite a high prevalence, therapies remain palliative. Therefore, it is of great interest to develop new approaches to curatively treat ADDE. Mesenchymal stem/stromal cells (MSC) have been shown to induce tissue regeneration and cease inflammation. Moreover, an increasing amount of MSC was found in the regenerating LG of mice. Therefore, this study investigated the therapeutic effect of MSC transplantation on damaged LGs using duct ligation induced ADDE in mice. Due to the transplantation of sex-mismatched and eGFP-expressing MSC, MSC could be identified and detected until day 21. MSC transplantation significantly improved LG regeneration, as the amount of vital acinar structures was significantly increased above the intrinsic regeneration capacity of control. Additionally, MSC transplantation modulated the immune reaction as macrophage infiltration was delayed and TNFα expression decreased, accompanied by an increased IL-6 expression. Thus, the application of MSC appears to be a promising therapeutic approach to induce LG regeneration in patients suffering from severe DED/ADDE.

## Introduction

Dry eye disease (DED) is a multifactorial disease affecting the entire lacrimal functional unit (LFU) including the ocular surface, the lacrimal glands (LG), the meibomian glands, the nervous innervation and the lids. Collectively, the LFU produces the complex and multi-layered tear film required for maintaining a physiological ocular surface^1^. The major part of the tear film is composed of the aqueous lacrimal fluid, which is secreted by the LG. The functional tissue of the LG -the acini- consists of secretory acinar cells, duct cells and myoepithelial cells^2^. Any impairment or loss of functional LG tissue lead to an imbalance of tear film homeostasis and can result in the development of aqueous deficient dry eye (ADDE). This DED subtype causes the most severe courses of the disease. During disease progression, the imbalanced tear film results in ocular surface inflammation, which can lead to corneal ulcers, as well as conjunctival and corneal scars, and thus to impaired vision^2^. Despite a high prevalence of DED with 5–50% depending on ethnic group, age and sex^3^, current treatment options remain palliative. Therefore, it is clinically of great importance to develop new approaches for a causative therapy. *In situ* regeneration of functional LG tissue has emerged to be a promising approach, and current studies are investigating drugs, gene therapy and stem cell therapy to induce/enhance LG regeneration^4^. However, further research is needed to overcome certain limitations.

One promising source for stem cell therapy to induce LG regeneration might be the use of mesenchymal stem/stromal cells (MSC), as these cells can be isolated from many different adult tissues and have already shown to exert therapeutic effects on the regeneration of glandular tissues, like pancreas, salivary gland (SG) and LG with chronic DED^5–7^. In addition, MSC have also been identified and isolated from the healthy and diseased rodent LG^8–10^ and it was shown that the number of MSC increase in regenerating LGs after experimentally induced ADDE^11–13^. In recent years MSC have been extensively studied and it was found that MSC exert therapeutic effects in a variety of pathological conditions^14,15^. A huge body of evidence shows that the therapeutic effects of MSC rely on the ability to suppress inflammation and initiate endogenous repair mechanisms. Furthermore, it was shown that MSC secrete trophic factors that affect infiltrating immune cells as well as tissue resident stem cells^16,17^.

In general, tissue inflammation and new tissue formation followed by tissue remodelling are the three stages of endogenous tissue repair initiated after acute damage^18^. Investigations on mouse models with experimentally induced ADDE revealed that the dynamic of LG damage and regeneration passes through the same three phases of tissue repair^11,19^. As the first two phases include the action of infiltrating immune cells and tissue resident stem cells, which are a target of MSC secreted factors, one could argue that the therapeutic effects of MSC are most valuable when applied directly after acute damage and during the first phase of tissue regeneration.

Ligation of the single secretory duct of the extraorbital LG was identified to induce severe ADDE in mice^11,13^. Duct ligation (DL) caused a profound loss of functional LG tissue, a severe inflammatory reaction and a reduced tear secretion. The LG, like other glandular tissues, retains the ability of self-regeneration after acute damage throughout its life-time, although it can be impaired due to chronic pathological conditions^20^. For this reason, the re-opening of the duct in the DL mouse model initiated a phase of new tissue formation/regeneration in juvenile mice, shown by the partial regeneration of vital acinar structures after 21 days by our working group^11^. This regeneration process was accompanied by an increase of intrinsic MSC.

In this study, the therapeutic efficacy of MSC transplantation was investigated regarding LG regeneration after surgically induced ADDE in mice. This will allow to assess whether the transplantation of extrinsic MSC supports the regeneration of the LG and could be useful in a clinically relevant setting. The use of tissue-specific extrinsic MSC is of great clinical relevance as the LG of patients with severe ADDE exhibit an impaired regenerative potential due to chronic pathological conditions such as persistent inflammation as well as age-dependent degeneration. Since MSC can be found in a variety of tissues and tissue-specific differences between the sub-populations have been described^21–23^, the use of LG-specific MSC for the treatment of ADDE seems to be superior to treatment with ectopic MSC. Consequently, MSC were isolated from murine LG of male mice expressing eGFP and characterized according to the defined minimal criteria^24^. DL was implemented on female mice for three days and eGFP-MSC were transplanted when releasing the DL. The analysis of vital acinar structures as the functional tissue of the LG at different time points after duct re-opening (day 5 and day 21) revealed that the transplantation of extrinsic MSC led to an enhanced increase in vital tissue area compared to saline injected LGs. This study provides the first evidences of a regenerative effect of extrinsic tissue specific MSC in an ADDE mouse model.

## Results

### Characterization of eGFP-MSC

**Figure 1:**
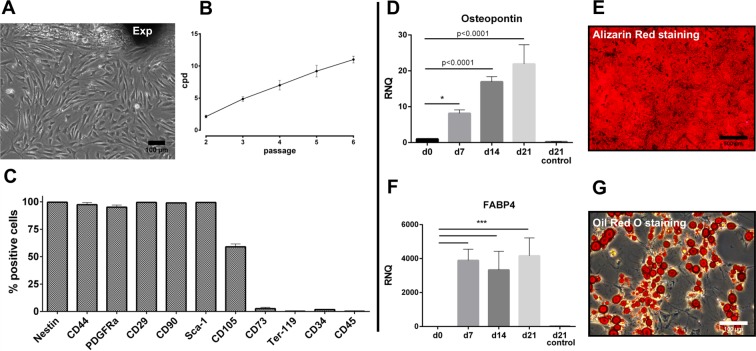
Characterization of eGFP-MSC. (**A**,**B**) eGFP MSC emerging from the explant (Exp) exhibited a spindle-shaped, fibroblastic morphology with a closely linear growth behavior, as detected by the assessment of cpd. (**C**) Immunophenotyping in p2 revealed the expression of typical MSC markers, while common hematopoietic (stem) cell markers were absent. Two distinct populations were detected for CD105. (**D**,**E**) The ability to differentiate towards osteocytes was verified by osteopontin expression and Alizarin Red staining. (**F**,**G**) Adipogenic differentiation was confirmed by FABP4 expression and Oil Red O staining. The quantity of positive cells [%] after isotype control staining are provided in the supplementary data file as Fig. S1. Data are n = 3, mean ± SD; scale bar: 100 µm (**A**,**G**) scale bar: 500 µm. (**E**) *Represents p ≤ 0.05 and ***represent p ≤ 0.001 compared to d0 control.

To verify the phenotype of MSC isolated from genetically modified eGFP-mice, the cells were characterized according to the defined minimal criteria^24^. Cells emerging the LG explant (Exp) exhibited a spindle-shaped, fibroblastic morphology (Fig. 1A). The cumulative population doublings (cpd) revealed a closely linear growth behavior up to passage (p) 6 (11.0 ± 0.53 cpd) as the coefficient of determination (r^2^) was 0.9707 (Fig. 1B).

eGFP-MSC expressed the commonly analyzed markers, as the population was >95% positive for nestin, CD44, PDGFRα, CD29, CD90, Sca-1 and <5% for CD73, Ter119, CD34 and CD45 (Fig. 1C). Two CD105 populations were detected, with 59.0 ± 2.6% of MSC being CD105-positive.

Differentiation capacity towards osteocytes and adipocytes was determined. After induction of osteogenesis, the expression of osteopontin significantly increased over time compared to control groups (p = 0.0187 at d7 and p < 0.0001 at d14 and d21; Fig. 1D). In addition, Alizarin Red staining exposed the mineralization of calcium-phosphate deposits and confirmed the differentiation (Fig. 1E). Progression of adipogenesis was monitored by FABP4 expression, which significantly increased after induction compared to control groups (p < 0.0001 at all investigated time points; Fig. 1F). Furthermore, formation of lipid droplets was visualized by Oil Red O staining (Fig. 1G).

### Investigation of eGFP-MSC for transplantation

**Figure 2:**
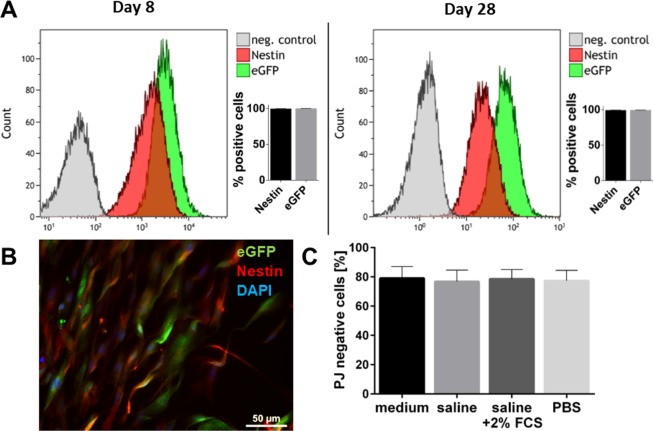
Evaluation of eGFP-MSC for Transplantation. (**A**,**B**) Nestin expression in eGFP-MSC was detected by flow cytometry and immunostaining. (**A**) A stable expression of nestin and eGFP was verified over 28 days. (**C**) Incubation of eGFP-MSC in culture medium, saline, saline +2%FCS or PBS for 3–5 hours on ice followed by treatment with a 27-gauge needle showed that approx. 80% of eGFP-MSC remained vital (PI negative). Data are n = 3, mean ± SD; scale bar: 50 µm.

To assess the genetical stability of eGFP-MSC concerning their stemness, we next investigated nestin, a commonly used marker for multipotent stem cells^25^, and the genetically expressed eGFP, as a tracking marker. The eGFP-MSC expressed nestin, which was previously shown to be expressed by MSC isolated from wild-type (wt) B6 LG (Fig. 2A,B)^26^. In addition, nestin and eGFP expression was stable (>99% each) over 28 days (Fig. 2A). These time interval correlates with the last examination time eGFP-MSC in the transplanted LGs (7 days in culture (*in vitro*) and a maximum of 21 days after transplantation (*in vivo*)).

To investigate the influence of different solution, which can be used for transplantation in mouse surgery, on the vitality of MSC, the cells were incubated on ice for 3–5 h in either culture medium, saline, saline +2% FCS, or PBS and then treated once with a 27-gauge needle to mimic transplantation. Propidium iodide staining in flow cytometry showed that, regardless of the solution used, about 80% of the MSC remained vital (Fig. 2C).

### Clinical assessment of ADDE

**Figure 3:**
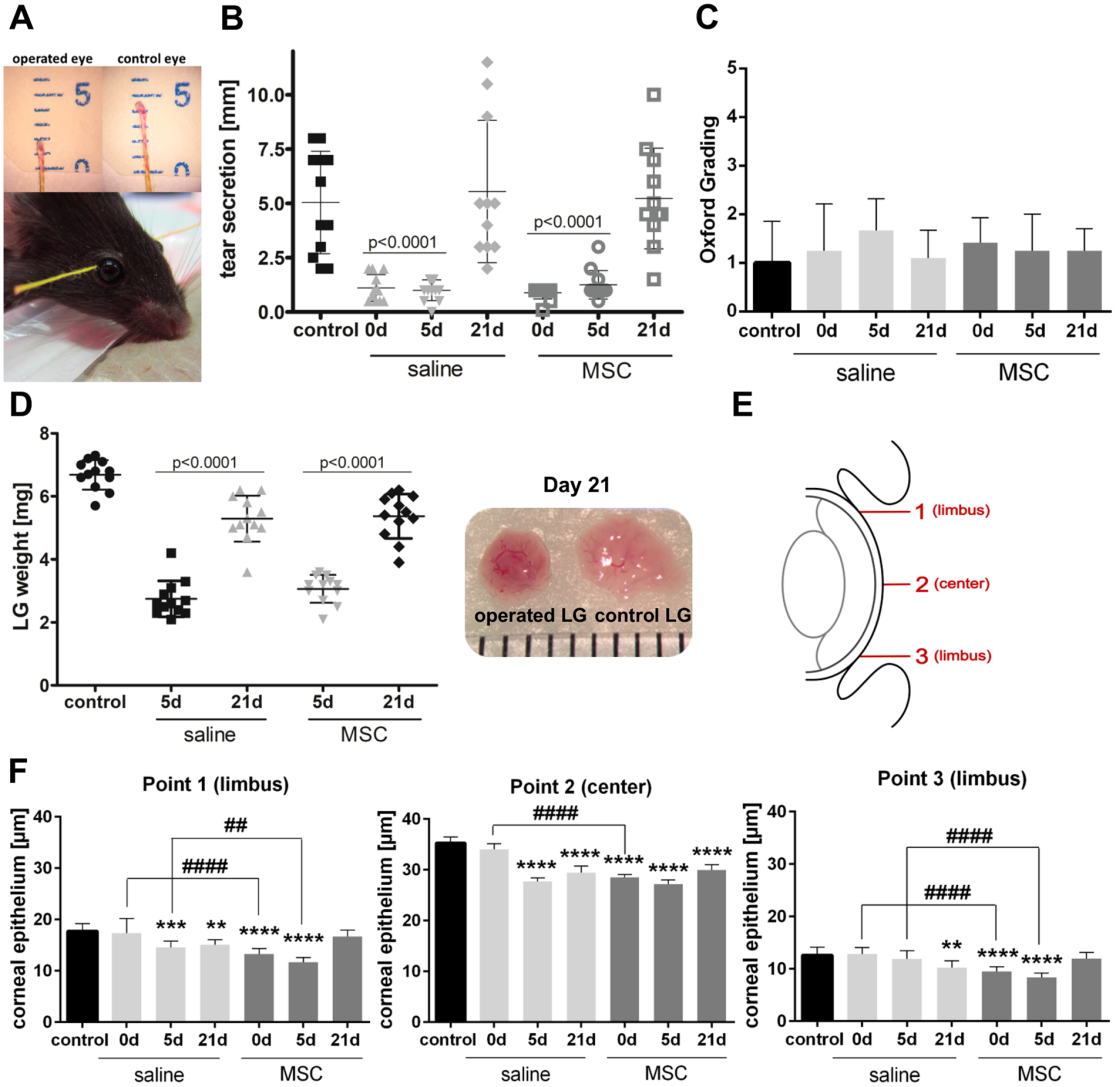
Clinical Assessment of ADDE. (**A**) Phenol red cotton threads were applied to the lateral canthus of each eye and the wetted distance was measured. (**B**) Tear secretion was significant reduced after DL (0d) but recovered to control levels by d21. (**C**) Fluorescein staining showed no differences at any time after DL. (**D**) LG weight was significantly reduced at d5 after DL and saline or MSC injection. From d5 to d21 LG weight significantly increased in both groups. (**E**) Measuring points were defined to cover the whole cornea (**F**) Thickness of corneal epithelium was significantly decreased after DL at almost any time and in any area of the ocular surface after saline and MSC injection compared to control. Data are n = 12 (**B**–**D**), n = 6 (**F**), mean ± SD; **represents p ≤ 0.01, ***represent p ≤ 0.001 and ****represent p ≤ 0.0001 compared to control; ^##^represents p ≤ 0.01 and ^####^represent p ≤ 0.0001 compared between saline and MSC groups.

To verify the induction of ADDE after DL clinically relevant measurements were performed^27^. The major impact of ADDE is the reduced secretion of lacrimal fluid onto the ocular surface, therefore tear secretion was assessed by a Schirmer test using phenol red cotton threads (Fig. 3A). DL resulted in a tear secretion of 1.10 ± 0.62 mm after saline injection and 0.88 ± 0.29 mm after MSC transplantation (d0), which was significantly reduced compared to basal secretion of 5.04 ± 2.36 mm (Fig. 3B). At day 5 tear secretion was still significantly reduced in both groups (p < 0.0001). However, 21 days of regeneration led to a significant increase with 5.55 ± 3.28 mm secreted tears after saline injection and 5.23 ± 2.32 mm after MSC transplantation, which was comparable to basal secretion.

The presence of fluorescein staining of the ocular surface is a well-established clinical method to measure severity of DED^2,27^, as fluorescein stains the corneal stroma when the epithelium is disrupted. Therefore, the integrity of the corneal epithelium was investigated by fluorescein staining (Fig. 3C). The evaluation showed no differences neither between control and treatment groups, nor between MSC and saline injection.

Damage and regeneration of LGs were further investigated by measuring the LG weight after excision (Fig. 3D). 5 days after re-opening of DL the LG weight was significantly reduced to 2.75 ± 0.57 mg in saline injected and to 3.07 ± 0.44 mg in MSC transplanted LGs compared to control (6.68 ± 0.47 mg). Regeneration of LG up to day 21 led to a significant increase in weight in saline and MSC injected LGs, respectively (p < 0.0001). This resulted in a LG weight comparable to control.

To further determine whether DL had an impact on the ocular surface, thickness of the corneal epithelium was assessed (Figs. 3E,F and S2). Thickness of corneal epithelium decreased significantly at almost any time and in any area after saline and MSC injection compared to control. In the limbal area, only on day 0 after saline injection and on day 21 after MSC transplantation, values comparable to control were observed. Additionally, in the limbal region the thickness of the corneal epithelium was significantly lower after MSC transplantation than after saline injection on day 0 and day 5.

### Assessment of MSC after transplantation

**Figure 4:**
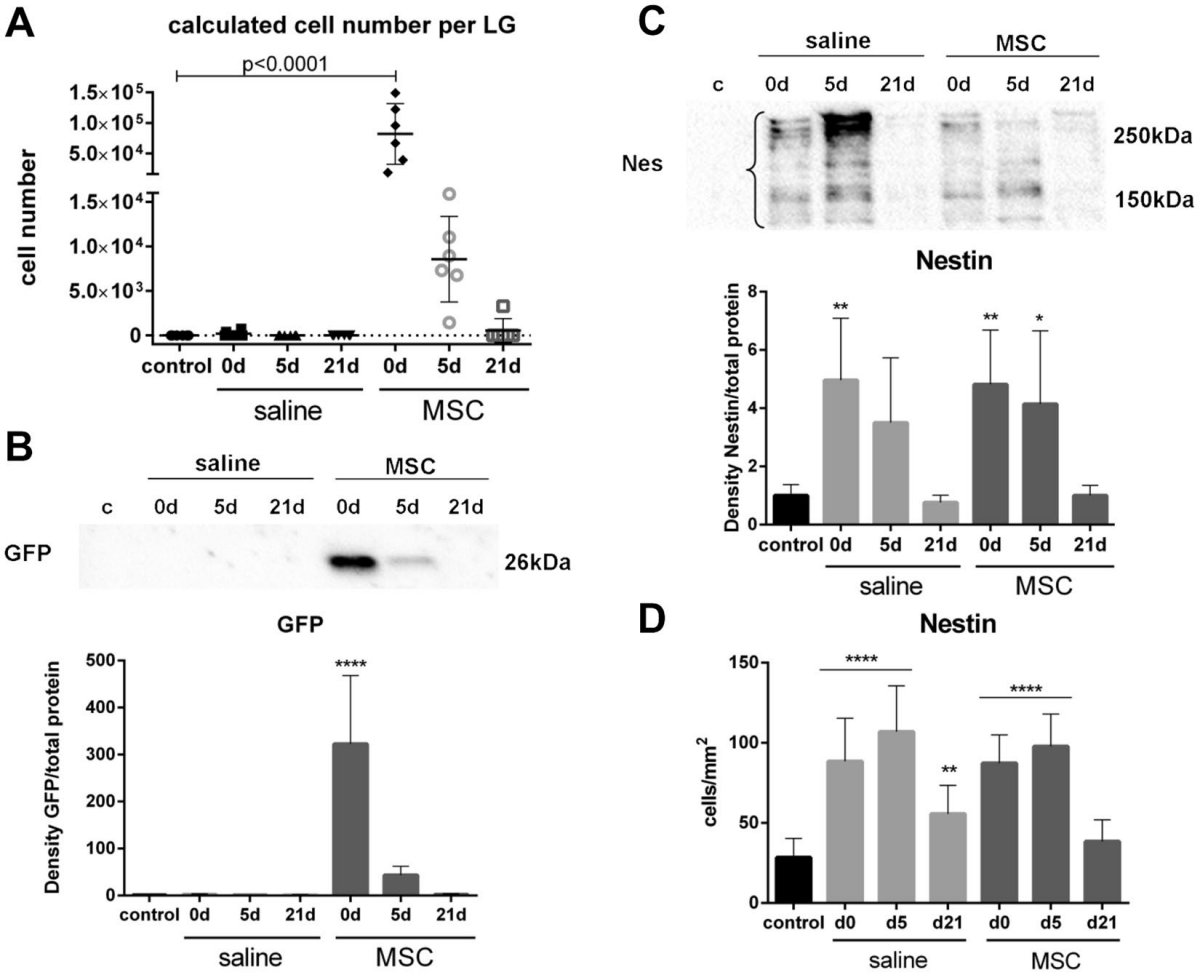
Assessment of MSC after Transplantation. (**A**) The number of transplanted cells was calculated according to the presence of male gDNA in female recipient gDNA, by *Rbmy* expression in qPCR. Five min (d0) after transplantation the number of MSC were significantly increased and then gradually returned to control. (**B**) Representative pictures of eGFP detection by western blot. Quantification revealed a significant increase 5 min (d0) after MSC transplantation, which decreased to control levels by d21. (**C**) Representative pictures of nestin detection by western blot. Nestin was significantly increased in saline and MSC injected LG at d0 and d5 compared to control. (**D**) Immunohistochemical staining detected nestin cells, which were significantly increased in saline and MSC injected LG at d0, which further increased at d5. At d21 the number of nestin cells was still significantly increased in saline, but not in MSC group. For western blot analysis the samples (n = 42) were run on four blots, which were processed in parallel. Full blots are provided in the supplementary data file. Data are n = 6, mean ± SD; *represents p ≤ 0.05, **represents p ≤ 0.01 and ****represent p ≤ 0.0001 compared to control.

To track the MSC within the injured LGs, transplantation of MSC from male eGFP-mice into LGs of female recipients (both mouse strains had the BL/6J background) was performed. The number of MSC present in LG was calculated based on male gDNA (MSC) in female gDNA (recipient mice) using qPCR amplification of a male-specific sequence (*Rmby*). Five min after transplantation 8.19 × 10^4^ ± 4.98 × 10^4^ MSC were detected, which gradually decreased to 5.51 × 10^2^ ± 1.33 × 10^3^ MSC at day 21 (Fig. 4A).

To confirm the presence of MSC, intrinsic eGFP was quantified. The results showed that the amount of eGFP protein was significantly increased (p < 0.0001) 5 min after transplantation, relative to control and saline injection (Fig. 4B). During regeneration the amount of eGFP decreased and was comparable to that of control at day 21 (relative, normalized density eGFP: 2.12 ± 1.76, control: 1.0 ± 0.51). In immunohistochemical staining, eGFP cells were detected in the stroma adjacent to acinar structures and could be found 5 min, 5 days and 21 days after transplantation (Fig. S3).

To investigate the presence of intrinsic and transplanted MSC during regeneration, nestin expressing cells were studied. Due to DL (d0) the amount of nestin was significantly increased (p = 0.004 for saline and p = 0.006 for MSC) relative to control (Fig. 4C). At day 5 after re-opening of DL and injection of saline, the amount of nestin was still, albeit not significantly, increased. Whereas, at day 5 after MSC transplantation the amount of nestin was still significantly increased (p = 0.037) relative to control. By day 21 the amount of nestin was comparable to control in both groups. In immunostaining, nestin expressing cells appeared as elongated, fibroblastic cells in the stroma of (damaged) LGs (Fig. S4). Number of nestin-positive cells were significantly increased after DL with 88.4 ± 26.88 cells/mm^2^ in saline and 87.33 ± 17.64 cells/mm^2^ in MSC injected LGs compared to control (28.4 ± 11.87 cells/mm^2^; Fig. 4D). The number of nestin positive cells increased to 106.9 ± 28.6 cells/mm^2^ in saline and 97.76 ± 20.18 cells/mm^2^ in MSC injected LGs at day 5. Although the number of nestin cells decreased by day 21, it was still significantly increased when saline, but not MSC, was injected compared to control. These findings were additional confirmed by analysis of nestin expression in qPCR (Fig. S5).

### Dynamic of LG damage and regeneration

**Figure 5:**
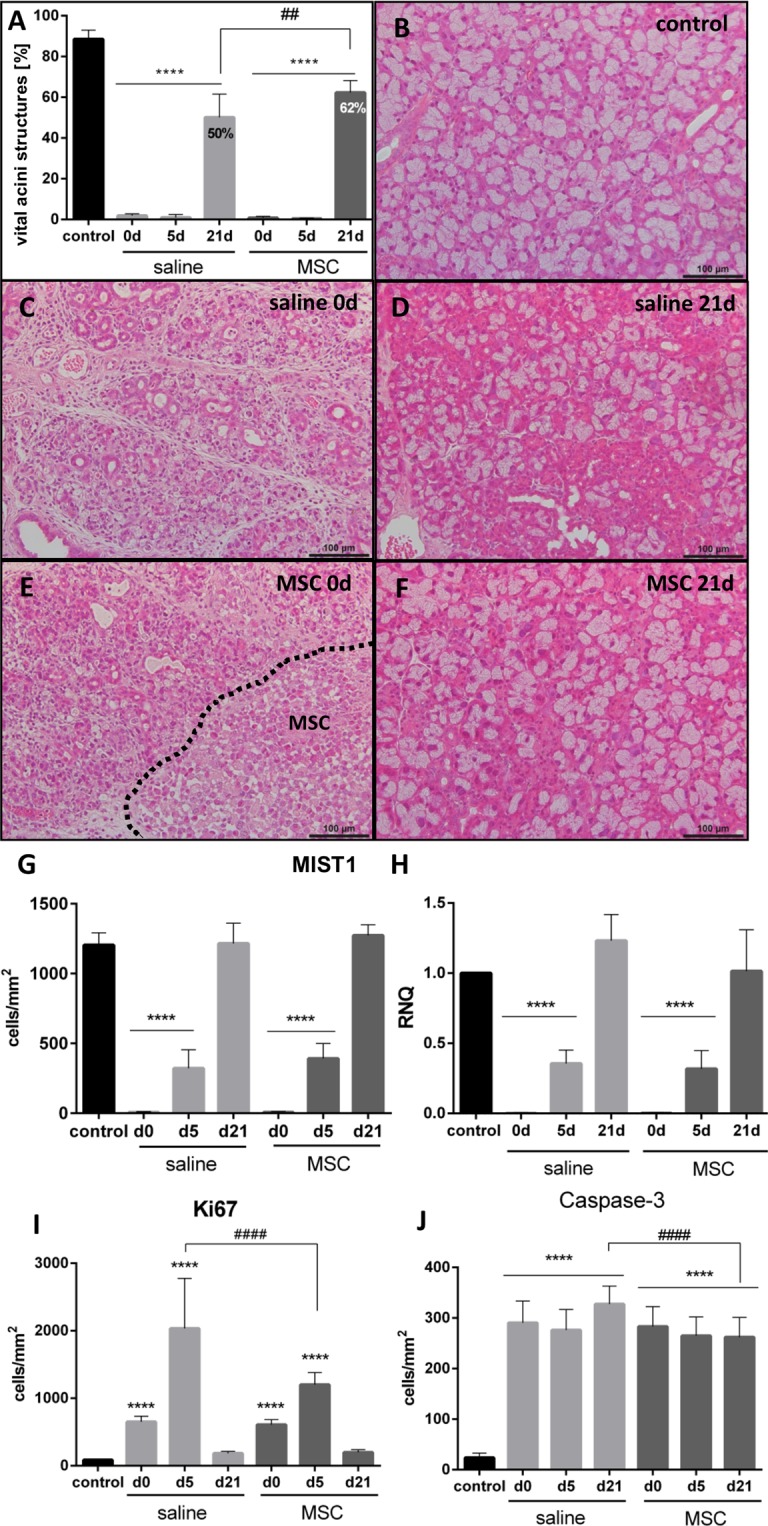
Dynamic of LG damage and regeneration. (**A**) Area of vital acinar structures calculated in HE stained LG sections. At d21 vital acinar structures recovered to significant higher extent after MSC than saline injection. (**B**) Control LG had tightly arranged acini, which were organized in lobules and surrounded by few connective tissue. (**C**,**E**) Due to DL, LG structure was damaged by interstitial edema, infiltrating cells and shrunken acinar cells with higher eosinophilia. (**E**) Transplanted MSC were detected in the stroma adjacent to acinar structures (dashed line). (**D**,**F**) After re-opening of DL and regeneration, LG structure recovered, and acinar structures re-appeared. (**G**) The number of MIST1-positive cells in immunohistochemical staining significantly decreased after DL (d0), but gradually increased thereafter and was comparable to control by d21. (**H**) MIST1 gene expression displayed comparable results to MIST1 immunohistochemical staining. (**I**) The number of Ki67-positive cells in immunohistochemical staining gradually increased up to d5 and normalized comparable to control at d21. At d5 the number of Ki67-positive cells were significantly higher after saline injection than after MSC injection. (**J**) The number of caspase-3-positive cells in immunohistochemical staining was significantly elevated at all time points in both groups. The number of caspase-3 positive cells at d21 was significantly elevated in saline injected LGs compared to MSC injected LGs. Data are n = 6, mean ± SD; scale bar: 100 µm. ****Represent p ≤ 0.0001 compared to control; ^##^represents p ≤ 0.01 and ^####^represent p ≤ 0.0001 compared between saline and MSC groups.

LG damage and regeneration were investigated in HE stains, and vital acinar structures were identified and measured (Fig. 5A–F). In control LG, the acinar structures are tightly arranged, and only sparse connective tissue was found between the lobules (Fig. 5B). DL resulted in interstitial edema, infiltrating cells and shrunken acinar cell body with increased eosinophilia (Fig. 5C,E). Transplanted MSC could be detected in the stroma adjacent to the acinar structures (Fig. 5E dashed line, Fig. S4). Re-opening of the DL resulted in the reappearance of acinar structures and a decline of interstitial edema (Fig. 5D,F). Analysis revealed that vital acinar structures significantly decreased from 88.58 ± 4.38% in control to 1.86 ± 0.96% and 0.81 ± 0.69% in saline and MSC injected LGs, respectively (d0, Fig. 5A). Five days after saline injection and MCS transplantation the area of vital acinar structures was still significantly decreased (p < 0.0001). During 21 days of regeneration, vital acinar structures increased to 62.32 ± 5.88% after MSC transplantation, which was significantly enhanced compared to saline injection (50.11 ± 11.45%, p = 0.0039).

Analysis of MIST1, an acinus specific transcription factor, confirmed the loss of acinar cells during DL (Figs. 5G and S6A). The number of MIST1-positive cells decreased from 1205 ± 87.55 cells/mm^2^ in control to 6.27 ± 5.42 and 6.93 ± 6.32 cells/mm^2^ in saline and MSC injected LGs, respectively. MIST1-positive cells increased gradually and were comparable to control by day 21 with 1216 ± 145.1 and 1272 ± 77.21 cells/mm^2^ after saline injection and MSC transplantation, respectively. The absence of MIST1 expression after DL was further confirmed by qPCR (Fig. 5H).

Damage and regeneration were further investigated by proliferating (Ki67) and apoptotic (caspase-3) cells. The number of Ki67-positive cells gradually increased after DL, up to day 5 to 2033 ± 742 cells/mm^2^ after saline injection and to 1201 ± 180 cells/mm^2^ after MSC transplantation (Figs. 5I and S6B), which was significantly more Ki67-positive cells in saline than in MSC injected LGs (p < 0.0001). The number of Ki67-positive cells normalized to control levels by day 21. The number of caspase-3-positive cells was significantly increased at all investigated time points and in both groups (p < 0.0001, Figs. 5J and S6C). However, at day 21 the number of caspase-3 cells was significantly lower after MSC transplantation than after saline injection (p < 0.0001).

### Characterization of immune reaction

**Figure 6:**
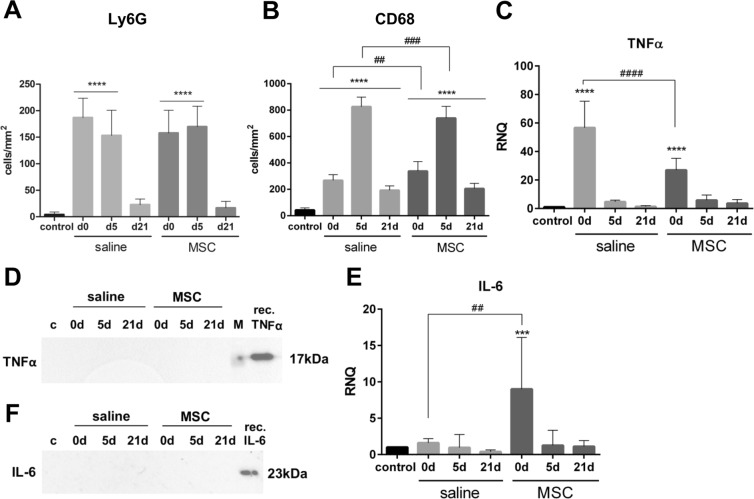
Characterization of immune reaction in LG. (**A**) Number of Ly6G cells in IHC significantly increased after DL in saline and MSC injected LGs at d0 and d5 but normalized by d21. At d0 the number of Ly6G cells tended to be higher after saline injection than MSC injection. (**B**) Number of CD68 cells in IHC was significantly increased at all time points compared to control. The number of CD68 cells differed significantly between saline injection and MSC transplantation at d0 and d5. (**C**) TNFα expression in the LG was significantly increased after DL (d0) and was significantly higher after saline injection than after MSC injection. (**D**) TNFα could not be detected in western blot. (**E**) Expression of IL-6 in the LG was increased only at d0 after MSC injection, which was significantly different compared to saline injection. (**F**) IL-6 could not be detected in western blot; for western blot analysis the samples (n = 42) were run on four blots, which were processed in parallel. Full blots are provided in the supplementary data file. Data are n = 6, mean ± SD. ***Represent p ≤ 0.001 and ****represent p ≤ 0.0001 compared to control; ^##^represents p ≤ 0.01, ^###^represent p ≤ 0.001 and ^####^represent p ≤ 0.0001 compared between saline and MSC groups.

To investigate the immune reaction after DL, we performed immunostaining and western blot analysis of commonly infiltrating cell types and pro-inflammatory cytokines. Number of Ly6G-positive cells, a marker for (neutrophil) granulocytes, was significantly increased from 4.1 ± 4.8 cells/mm^2^ in control to 186.9 ± 36.8 cells/mm^2^ and to 157.7 ± 43.2 cells/mm^2^ after saline and MSC injection (d0), respectively (Figs. 6A and S6D). Number of infiltrating Ly6G-positive cells remained high at day 5 in both groups (p < 0.0001, respectively), but decreased to control levels at day 21.

Number of CD68-positive cells, a marker for monocytes and macrophages, was significantly increased at all investigated time points, both in saline and MSC injected LGs, compared to control (p < 0.0001, Figs. 6B and S6E). The number of CD68-positive cells increased from 43.1 ± 16.5 cells/mm^2^ in control to 268.3 ± 42.8 cells/mm^2^ after saline injection and to 228.2 ± 72.3 cells/mm^2^ after MSC transplantation (d0), which was significantly different when comparing the two treatment groups (p = 0.0073). Infiltration of CD68-positive cells further increased at day 5 to 826.3 ± 72.3 cells/mm^2^ and to 739.7 ± 89.2 cells/mm^2^ after saline and MSC injection, respectively. At day 5 the number of CD68-positive cells was significantly lower in MSC than in saline injected LG (p = 0.0005). By day 21 the number of CD68-positive cells significantly decreased in both groups compared to day 5 but was still significantly higher than in control.

Gene expression of TNFα raised 56.7 ± 18.6-fold after saline injection and 26.95 ± 8.3-fold after MSC transplantation, which was significantly up-regulated compared to control (Fig. 6C). Expression of TNFα was significantly higher after saline injection than after MSC transplantation (p < 0.0001). During regeneration, the expression of TNFα decreased and reached control levels by day 21. Although the expression of TNFα increased, no TNFα protein could be detected (Fig. 6D). Expression of IL-6, a widely described immune-modulatory cytokine secreted by MSC, was significantly increased at day 0 after MSC transplantation, but not after saline injection when compared to control (p = 0.0007; Fig. 6E). Thus, the expression of IL-6 was significantly higher in MSC than in saline group (p = 0.0037). However, no IL-6 protein could be detected (Fig. 6F).

### Influence of MSC

**Figure 7:**
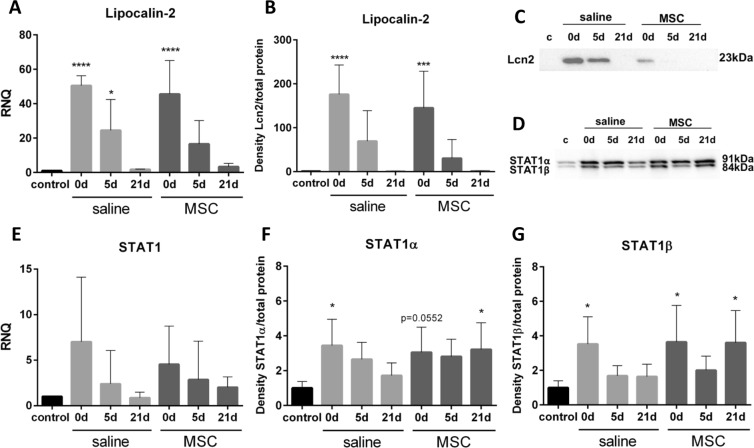
Influence of MSC. (**A**) Expression of Lipocalin-2 (Lcn2) significantly increased after DL (d0) and then gradually decreased to control level by d21 in both groups. (**B**,**C**) Western blot analysis of Lcn2. (**B**) Quantification of Lcn2 revealed a significant increase 5 min (d0), which decreased to control levels by d21 after saline and MSC injection. (**C**) Representative pictures of Lcn2 detection by western blot. (**D**) Representative pictures of STAT1α/β detection by western blot. (**E**) Expression of STAT1 slightly increased at d0 and d5. (**F**) Quantification of STAT1α revealed a slight increase at all time points after saline and MSC injection. (**G**) Quantification of STAT1β revealed a slight increase at all time points after saline and MSC injection. For western blot analysis the samples (n = 42) were run on four blots, which were processed in parallel. Full blots are provided in the supplementary data file. Data are n = 6, mean ± SD. *Represent p ≤ 0.05, ***represent p ≤ 0.001 and ****represent p ≤ 0.0001 compared to control.

In a previous study, we identified lipocalin-2 (Lcn2) and STAT1 in the secretome of MSC, contributing to the improvement of LG epithelial cell survival *in vitro*^26^. Therefore, we analyzed whether transplanted MSC also secrete Lcn2 and/or STAT1 *in vivo*. Expression of Lcn2 was significantly increased 5 min (d0) after saline and MSC injection compared to control (p < 0.0001) and gradually decreased to control levels by d21 (Fig. 7A). Investigation on Lcn2 protein level also revealed a significant increase at d0 (p < 0.0001 saline injection and p = 0.0004 after MSC transplantation) with a return to baseline by day 21 (Fig. 7B,C). Expression of STAT1 increased at d0 in both groups and gradually declined over time (Fig. 7E). Protein levels of STAT1α as well as STAT1β were significantly increased at d0 in saline and MSC injected LGs (STAT1α: p = 0.0127 saline, p = 0.0552 MSC; STAT1β: p = 0.0316 saline, p = 0.021 MSC) compared to control (Fig. 7D,F,G). In saline injected LG the level of STAT1α and STAT1β decreased thereafter. In MSC injected LG the amount of STAT1α and STAT1β declined by d5 and was again significantly increased at d21 compared to control (STAT1α: p = 0.0302, STAT1β: p = 0.0241).

## Discussion

ADDE, due to a loss of functional LG tissue, causes the most severe forms of DED^28^. Current therapies remain palliative and even advanced therapies, like the transplantation of the SG can only ease the symptoms, but are insufficient to restore the physiologic composition of the tear film and to address the underlying loss of LG tissue^29^. Therefore, this study evaluated a curative approach by investigating the therapeutic potential of LG-specific, extrinsic MSC transplantation to enhance LG regeneration in an experimental model of ADDE.

In the current study, MSC for transplantation were isolated by explant culture from mice that express eGFP in a constitutive and ubiquitous manner (eGFP-MSC). Recently, explant culture was identified as a suitable method to isolate a pure, specific and functional MSC population from the murine LG^8,26^. The eGFP expression allowed the identification and tracking of transplanted MSC throughout the study. Nevertheless, genetic modification and ectopic expression of a transgene (eGFP) might alter the physiology of the cells. Therefore, eGFP-MSC were characterized and compared to results obtained from wildtype (wt) mice in previous studies^8,26^. eGFP-MSC exhibited the characteristic fibroblastic morphology and growth behaviors comparable to that of wt-MSC^22,26,30^. In addition, phenotypic characterization and differentiation capacity was comparable to that of MSC from other tissues^22,31^ and to that of wt-MSC^8,26^. The genetic stability of eGFP-MSC was shown by a constant expression of nestin and eGFP over the entire timespan of the study (28 days). Overall, the results of the current study confirm that eGFP-MSC have the same properties than wt-MSC, can be tracked by a stable eGFP expression over 28 days and are therefore suitable for transplantation experiments.

A well-established model was used to induce severe ADDE in mice by ligation of the secretory duct of the LG, which has been shown to cause a profound loss of functional tissue in mice and rabbits, mimicking the tissue damage in patients with ADDE^11,13,32^. The key feature of ADDE in patients, is the abated tear secretion^2,27,33^. In the current study, a significant decrease of tear secretion confirmed the successful induction of ADDE, which was further proven by a loss of LG weight. In the course of ADDE, the reduced tear secretion causes damage to the ocular surface, which can be determined by a variety of dyes and a decreased thickness of corneal epithelium^2,27,34,35^. Although DL had no macroscopic impact on the ocular surface in the current study, visualized by fluorescein staining, the influence on the ocular surface was demonstrated by a decreased thickness of the corneal epithelium. Presumably, the period of reduced tear secretion in the mouse model was too short to cause a severe damage to the integrity of the corneal epithelium. In summary, the three-day DL resulted in clinical signs of acute ADDE with impaired functional LG tissue and tear physiology with minor impact on the ocular surface and can therefore be used as a model to study *in situ* LG regeneration.

Transplantation of MSC has emerged as a promising approach to induce regeneration of a variety of tissues and a lot of clinical trials were implemented^14,15,36^. A huge body of evidence indicates that MSC secrete trophic factors responsible for their induction of tissue repair. However, in addition to the secretion of trophic factors that affect tissue-resident progenitors or specialized tissue cells, it might be possible that MSC differentiate into the cells of the injured tissue and thereby replenish lost tissue^14^. In both cases, however, the engraftment of the MSC in the tissue of interest is a prerequisite for the successful induction of regeneration^16,37^.

One of the main influences on the homing efficiency of MSC in the tissue of interest is the site of MSC delivery. In murine LG studies bone marrow MSC were applied either systemically^5^ or periorbital^38^ and both resulted in improved LG function. Nevertheless, it is well known that cells can get trapped, e.g. in the lung, upon systemic transplantation^14,37^. Therefore, the present study performed a locally intra-glandular injection and verified the presence of MSC within the LG. An additional major advantage of the current study was the use of traceable MSC, which was achieved by sex-mismatched transplantation of MSC isolated from male eGFP-expressing mice. This allows the detection of engrafted MSC by both their male DNA and their eGFP expression^39^. Moreover, the detection of a male specific sequence, such as *Rbmy*, can be used to calculate the absolute number of engrafted MSC^40^. In the current study, tracking and calculation of MSC via *Rbmy* expression was done and their presence was further confirmed by the detection of eGFP in immunostaining. Accordingly, two independent tracking methods confirmed the presence of the transplanted, extrinsic MSC within the LG. The results showed that the number of MSC was high after transplantation and gradually decreased thereafter. Thus, transplanted MSC were present through acute inflammation of the LG and the initial phase of tissue repair.

The LG is composed of acinar, ductal and myoepithelial cells, which assemble into functional units (the acini). With approximately 80% of the cells, acinar cells represent the most important cell type of LG. Acinar cells produce and secrete the primary tear fluid which is a complex composition of inorganic salts, immunoglobulin A and various proteins such as lactoferrin, serum albumin, lysozyme and lipocalin^2,41^. The loss of vital acinar structures therefore leads to reduced quality and quantity of tear secretion and results in the development of ADDE. In this study we were able to prove that tissue specific MSC transplantation significantly improves the regenerative capacity of damaged acinar structures. MSC transplantation resulted in the recovery of vital acinar structures to 62% of total LG tissue, which is an increase of 25% compared to spontaneous regeneration after saline injection. The demonstrated improvement in regenerative capacity is of great clinical relevance as age-related degradation processes and chronic inflammation seems to affect the intrinsic LG regenerative capacity and therefore the initiation and restoration of LG regenerative capacity is highly desirable.

The enhanced amount of vital acinar structures after MSC transplantation confirms the high therapeutic potential of extrinsic LG-MSC and suggests that a therapeutic benefit can be achieved if the intrinsic regeneration potential is not sufficient to reinstate LG function. Our results are in line with recently published data, who showed a sustained improvement of tear secretion and a reduced infiltrated LG area after systemically bone-marrow MSC transplantation in NOD mice with autoimmune-mediated chronic DED^5^.

The investigation of the intrinsic and extrinsic MSC detected by their nestin expression showed a high number on day 0 and 5, but only a low number on day 21. Differences were observed between the treatment groups, as the number of nestin positive cells on day 21 was still significantly increased after saline injection but not after MSC transplantation. Since MSC exert their therapeutic effects mainly in the initial phase of tissue regeneration, the differences in MSC count in the groups indicate that the transplantation of extrinsic MSC leads to a shortening of the initial phase of regeneration. These findings further support that MSC transplantation is beneficial for LG regeneration.

One of the main underlying causes leading to the loss of functional LG tissue and thus to the development of ADDE, is inflammation^29^. A variety of studies showed that DL resulted in an acute inflammatory reaction in SG^42,43^ and LG^11,13^. In line with this, in our study a severe inflammatory reaction was also detected in form of a massive infiltration of neutrophils and macrophages at day 0 and day 5 and decreased at day 21. During this severe inflammatory reaction, the proportion of vital acinar structures is diminished and starts to regenerate when the inflammation decreases. Comparing the two treatment groups, the number of macrophages was higher at day 0 and lower at day 5 after MSC transplantation than after saline injection. This is presumably a result of the immune modulatory properties of transplanted MSC, since a variety of studies showed that MSC inhibit monocyte maturation and macrophage proliferation^16^. In general, it is assumed that modulation of the immune system is one of the key elements of MSC-mediated tissue repair^17^. One of the immunoregulatory factors secreted by MSC is IL-6, whose expression was highly upregulated upon MSC transplantation in this study. This indicates that the immunomodulation of transplanted MSC in this ADDE model also included the action of IL-6. Moreover, it was described that MSC inhibit the production/secretion of the TNFα^44,45^. This is in line with the findings of the current study, where a decreased TNFα expression was detected after MSC transplantation. Overall, the results show that the transplantation of MSC resulted in a modulation of the inflammatory reaction in the LG after DL.

The gradual loss of MSC over time in conjunction with the enhanced tissue regeneration indicates that the therapeutic effects of MSC rather rely on the secretion of trophic factors than on differentiation towards LG acinar cells. Indeed, studies of glandular tissues, others than the LG, detected that MSC-secretome or MSC-derived extracellular vesicles had a similar therapeutic effect, on the ability to maintain/restore glandular function, than the MSC itself^46–48^. Moreover, in recent studies it was shown that MSC conditioned medium had beneficial effects on injured LG epithelial cells *in vitro*^8,26^. Among others, Lcn2 and STAT1 could be identified in the secretome of MSC and were found to positively affect the viability of injured LG epithelial cells *in vitro*^26^. Therefore, the presence of these proteins was investigated in the current study.

Lcn2 expression and protein level were highly upregulated at day 0 and day 5 to comparable levels in saline and MSC injected LGs. As neutrophils also express Lcn2, it might be possible that the detected Lcn2 rather originated from the immune cells than from the implanted MSC^49^. This would coincide with the high numbers of neutrophils detected at day 0 in the current study and which were also comparable in both groups. This hypothesis is further supported by the results obtained from IL-1 induced ADDE, where Lcn2 was the most up-regulated gene at day 1 and day 2 and was accompanied by a massive neutrophil infiltration, whereas the number of MSC peaked at day 3^12,19^. Nevertheless, Lcn2 could have contributed to the regeneration of the LG as an overexpression in MSC was shown to increase their proliferative capacity, inhibit stress-induced apoptosis and induce expression of growth factors, such as HGF^50^. Consequently, Lcn2 could, in parallel to its direct effect on LG epithelial cells, influence the MSC by enhancing their regenerative effects and thus contribute to LG regeneration. Since there was a higher number of MSC in the LG directly after transplantation, the effects of Lcn2 on the MSC could have a greater impact on the MSC transplanted LG than on the saline injected LG. Consequently, Lcn2 might contribute to the improved LG regeneration after MSC transplantation.

The only difference in the protein level of STAT1 between saline and MSC injected LGs was found at day 21, where STAT1α/β was increased in MSC, but not saline injected LGs. In general, STAT1 signaling is complex and reveals somehow contrasting functions, which could be due to the large number of activating cytokines and receptors that signals through the JAK/STAT pathway as well as inducer-independent transcriptional activity of STAT1^51,52^. Known STAT1 functions include the promotion of apoptosis, regulation of tissue remodeling, but also the stimulation of progenitor cells proliferation^51,53–55^. Tissue remodeling after acute injury is complex and involves apoptosis of various cell types, and extracellular matrix (ECM) remodeling^18^. It has been shown that PDGF induced tissue remodeling, e.g. stimulated fibroblast proliferation, collagen secretion and increased ECM synthesis involve the activity of STAT1^56,57^. The elevated STAT1 protein level on day 21 after MSC transplantation might therefore indicate that the MSC-injected LGs may enter the third phase of tissue repair - tissue remodeling. However, further studies have to confirm whether tissue remodeling occurs in the regenerating LG as soon as day 21.

In conclusion, this study revealed that the transplantation of LG-specific MSC significantly improved the regenerative capacity of LG in an ADDE mouse model. The significantly improved and accelerated regeneration after MSC transplantation compared to saline injection was demonstrated by a significant increase of vital acinar structures, a shortened presence of MSC in the LG, earlier decline of apoptotic cells, a modulated macrophage invasion and a lower number of proliferating cells during acute inflammation, a lower expression of TNFα and an increased expression of IL-6. Thus, the use of extrinsic MSC appears to be a promising approach for the curative treatment of patients with severe DED/ADDE with impaired intrinsic LG regenerative capacity.

## Methods

### Mice

For transplantation experiments female C57BL/6J mice were obtained from Janvier labs (Le Genest-Saint-Isle, France). For the isolation of MSC, C57BL/6-Tg(CAG-EGFP)1Osb/J (eGFP) were purchased from the Jackson Laboratory (Sacramento, CA) and further bred in the Central Animal Facility (Heinrich-Heine-University, Duesseldorf, Germany). Mice were kept under 12:12 h light:dark cycle with food and water *ad libitum*. All experiments were implemented in accordance with the “Association for Research in Vision and Ophthalmology” Statement for the use of animals in ophthalmic and vision research, the national ethical committee for animal experimentation (FELASA guidelines) and were approved by the “Landesamt für Natur, Umwelt und Verbraucherschutz Nordrhein-Westfalen” (LANUV, IRB No. 84-02.04.2013.A268).

### Mesenchymal stromal/stem cell isolation

MSC were isolated from male eGFP-mice (8–12 wk). After euthanasia extraorbital LGs were placed in cold culture medium (α-MEM, 2 mM L-glutamine, 15% FBS-S (all purchased from Biochrom, Berlin, Germany) and 1% penicillin/streptomycin (Sigma-Aldrich, St. Louis, MO)). Before mincing with a scalpel, LGs were washed thrice with PBS (Sigma-Aldrich). Minced LGs were transferred to 10 cm culture dish and allowed to attach to the surface before culture medium was added.

### Characterization of mesenchymal stromal/stem cells

MSC were characterized according to the defined criteria^24^. LGs of 10 male eGFP-mice were used for each experiment.

Growth behavior and morphology were evaluated by inverse microscope observation and assessment of the cumulative population doubling (cpd) up to passage (p) 6.

Immunophenotyping was performed in p2 by using a CyAn ADP Flowcytometer (Beckman Coulter, Brea, CA), recording 10,000 viable single cells. In brief, detached cells were pre-incubated with anti-CD16/CD32 (BD Bioscience, San Jose, CA) for 5 min on ice. Thereafter, primary antibodies (see Table S1) were added and incubated for 30 min on ice. If intracellular staining was necessary, cells were fixed in 2% PFA for 30 min and permeabilized with 0.1% Triton-X-100 for 5 min and then proceeded as described above. Staining with the respectively labelled isotypes served as a control. Kaluza Software (Beckman Coulter) was used for analysis.

*In vitro* differentiation toward adipocytes and osteocytes was investigated in p3. In brief, 2500 cells/cm^2^ were plated onto a 6 cm culture dish. Differentiation was induced when cells reached 70% confluency (osteogenesis) or 90% confluency (adipogenesis) by replacing the culture medium with osteogenic or adipogenic induction medium (see Roth *et al*.^8^). Differentiation was assessed after 7, 14 and 21 days by investigating the expression of osteopontin for osteogenesis or fatty-acid binding protein 4 (FABP4) for adipogenesis through qPCR. In addition, differentiation was visualized by staining with Alizarin Red S (Sigma-Aldrich) for osteogenesis and Oil-Red O (Sigma-Aldrich) for adipogenesis at day 21. Staining was performed according to manufacturer’s instructions. Cells grown in culture medium served as control.

### Surgery

Mice (8–10 wk) were anesthetizes using ketamine (80 mg/kg bodyweight (BW); Zoetis, Florham Park, NJ) and xylazine (7.5 mg/kg BW; Bayer, Leverkusen, Germany). Right extraorbital LG was exposed and the associated duct was ligated twice using a silicon tube (AS ONE Corporation, Osaka, Japan; see video 2 in Dietrich *et al*.^11^). After 3 days, duct ligation (DL) was removed by reopening the silicon nodes and 2.5 × 10^5^ MSC in 2 µl saline were injected into the LG using a 27-gauge needle (Hamilton syringe). Injection of saline (2 µl) served as control. For transplantation experiments MSC were harvested 7 days after isolation. For analgesia, buprenorphine (0.05 mg/kg BW; Reckitt Benckiser, Slough, UK) was injected subcutaneously and tramadol (Hexal, Holzkirchen, Germany) added to the drinking water for 3 days at 1 mg/ml. The stitched wound was covered with Gentamicin ointment (5 mg/g, Ursapharm, Saarbrücken, Germany).

### Experimental setup

Samples were collected 5 min, 5 days and 21 days after MSC transplantation or saline injection. Fluorescein staining and tear production was measured on the three time points (n = 12/time). For histological analysis, mouse eyes and LGs were excised (n = 6/time) and fixed with 4% PFA. For molecular biologic assessment, LGs (n = 6/time) were snap frozen in liquid nitrogen and RNA, DNA and protein purification was performed using the AllPrep DNA/RNA/Protein Kit (Qiagen, Hilden, Germany) according to manufacturer´s manual.

### Fluorescein staining of the ocular surface

Fluorescein staining was performed by applying 5 µl fluorescein (1.7 mg/ml Fluorescein SE, Alcon, Freiburg, Germany) to the lateral cantus of each eye. Excess solution was removed with PBS (Sigma-Aldrich). Eyes were examined using a slit lamp with a cobalt blue filter (PSL Classic, Keeler, Windsor, UK) and the defects were classified according to the Oxford Grading System.

### Tear production

Tear production was measured using phenol-impregnated cotton threads (Zone-Quick, FCI Ophthalmics, Pembroke, MA). The threads were applied to the lateral canthus of both eyes for 60 s using forceps. In contact with tears the threads turned red and tear production was measured in millimeters under a dissecting microscope (Carl Zeiss, Oberkochen, Germany; Fig. 3A).

### HE staining

To evaluate the thickness of the corneal epithelium, paraffin embedded sections were stained with hematoxylin and eosin (HE). Three slides, and three sections per slide, of the middle eye at a distance of 10 slides were used. Sections were photographed with a microscope (DM4000B, Leica Microsystems, Wetzlar, Germany), merged for an overview image and measured using Fiji software^58^. To compare different areas of the cornea, three measuring points were defined: upper limbus (1), lower limbus (3) and the measured center between the two points (3, Fig. 3E).

For evaluation of LG structure and acini arrangement, pictures of HE stained whole LG were investigated using Fiji software^58^ and analyzed as previously described^11^. In brief, vital and severely affected acini structures were measured, and each proportion calculated based on total LG area (set as 100%). Severely affected acini were identified by a shrunken cell body with eosinophilia and irregular arrangement of acini structures.

### Immunohistochemistry

One central section (4 µm) was used for DAB staining as described before^11^. In brief, deparaffinized and rehydrated sections were subjected to antigen retrieval in 10 mM citrate buffer (pH 6.0) for 35 min in a steam oven. Thereafter, sections were permeabilized with 0.15% Triton-X-100 for 10 min, followed by endogenous peroxidase quenching with 3% hydrogen peroxide for 30 min. Unspecific binding was blocked using 25% equine serum (Sigma Aldrich) with/without 0.25% BSA. For mouse-on-mouse staining, the sections were pre-incubated with Fab-Fragment for 1 h (Table S1). Primary antibodies were applied overnight at 4 °C (Table S1). Sections were then incubated with the corresponding biotinylated secondary antibody (Table S1) before immunoreactivity was detected using an avidin/peroxidase system with 3,3′-diaminobenzidine (DAB) substrate. Positive stained cells were counted in 20-fields of view using a graticule (grid 10 × 10 mm) in 400-fold magnification using a microscope (DM4000B, Leica) and cells per mm^2^ were calculated.

### Immunocytochemistry

MSC grown on slides were fixed using 4% PFA for 15 min, permeabilized with 0.1% Triton-X-100 for 5 min before unspecific binding was blocked with 0.5% BSA. Primary antibodies were applied overnight at 4 °C (Table S1). Cells were washed with PBS and incubated with the corresponding fluorescence-labelled secondary antibody (Table S1).

### Quantitative real-time PCR (qPCR)

Amplification, including melting curve, was performed on a 96 well qPCR system (7500 Fast, Applied Biosystems). Duplicates of the six biological replicates were introduced for amplification with Power SYBR Green Master Mix (Applied Biosystems). No-template-samples served as controls. Expression was normalized to ribosomal protein S6 (RPS6) as endogenous control. Data analysis was performed according to Pfaffl and calculated as normalized relative fold expression level (RNQ)^59^. Primer sequences are listed in the Supplementary Table S2.

For the assessment of MSC engraftment male genomic (g)DNA was analyzed in a 20 µl real-time PCR. Primer pairs against TATA-box binding protein (TBP) were used as endogenous control and against RNA-binding motif protein on Y chromosome (*Rbmy*) to detect male-specific DNA (Table S2). Male mouse gDNA standards were generated adding 100, 50, 1, 0.5, 0.1, or 0.05 ng male gDNA to 100 ng female gDNA and were run along with the samples. To generate the standard curve, delta threshold cycles (CT) of standards were plotted against the concentration. Calculation of male gDNA per 100 ng introduced gDNA was performed according to Hong *et al*.^40^. In brief, it is assumed that a mouse genome contains 3.4 × 10^9^ base pairs (bp), leading to 6.8 × 10^9^ bp for a diploid genome. In addition, a double-stranded DNA bp has a molecular weight of 645 Daltons (D), and 1 D is equal to 1.65 × 10^−24^ g. Consequently, the DNA content of a diploid mouse cell is 7.2 pg (6.8 × 10^9^ bp × 645 D × 1.65 × 10^−24^ g).

### Western blot

Protein samples (15 µg) were denatured and separated on sodium dodecyl sulfate-polyacrylamide gels, followed by semi-dry transfer to nitrocellulose membranes (Amersham, GE Healthcare, Chicago, IL). Membranes were blocked with 5% skim milk powder or 5% BSA in Tris-buffered saline. Primary antibodies were diluted in blocking buffer and incubate at 4 °C overnight (Table S1). Horse-radish peroxidase (HRP) conjugated secondary antibodies were incubated for 1 h at room temperature. Chemiluminescence was developed using WesternBright Sirius HRP substrate (advansta, San Jose, CA) according to the manufacture’s instruction and visualized using the ChemiDoc MP Imaging system (Bio-Rad, Hercules, CA). For positive controls 40 ng recombinant murine IL-6 or TNFα (Peprotech, Rocky Hill, NJ) were run along with the samples. Total protein was stained with SYPRO Ruby according to the manufacturer’s instructions (Invitrogen, Waltham, MA) and used for normalization. Analysis was performed using ImageLab 6.0.1 (Bio-Rad, Hercules, CA). Images of all blots can be found in Supplemental Material.

### Statistics

GraphPad Prism 6 software (La Jolla, CA) was used for statistical data analysis. Values were given in means ± standard deviation (SD). Statistical analysis was performed using ANOVA with Tukey or Dunnet post-hoc test. Differences with p ≤ 0.05 were considered as significant.

## Supplementary information

Supplemental Material

## Data Availability

All data generated or analyzed during this study are included in this article, its supplementary information file or are available from the corresponding author on reasonable request.
