# Supplementary material for: MSC Transplantation Improves Lacrimal Gland Regeneration after Surgically Induced Dry Eye Disease in Mice
Source: Sci Rep. 2019 Dec 4;9:18299. doi: 10.1038/s41598-019-54840-5 (PMC6892942; doi:10.1038/s41598-019-54840-5)

Supplemental Material

MSC Transplantation Improves Lacrimal Gland Regeneration after Surgically Induced Dry Eye Disease in Mice

Jana Dietrich[1*], Lolita Ott[2], Mathias Roth[2], Joana Witt[2], Gerd Geerling[2], Sonja Mertsch[1], Stefan Schrader[1]

[1]Laboratory of Experimental Ophthalmology, Department of Ophthalmology, PIUS-HOSPITAL, Carl-von-Ossietzky University, 26121 Oldenburg, Germany

[2]Laboratory of Experimental Ophthalmology, Department of Ophthalmology, University of Duesseldorf, 40225 Duesseldorf, Germany

*Corresponding author:
Jana Dietrich, Master of Science
Department of Ophthalmology
PIUS-HOSPITAL, Carl-von-Ossietzky University,
Carl-von-Ossietzky Str. 9-11
26129 Oldenburg
E-Mail: jana.dietrich@uni-oldenburg.de

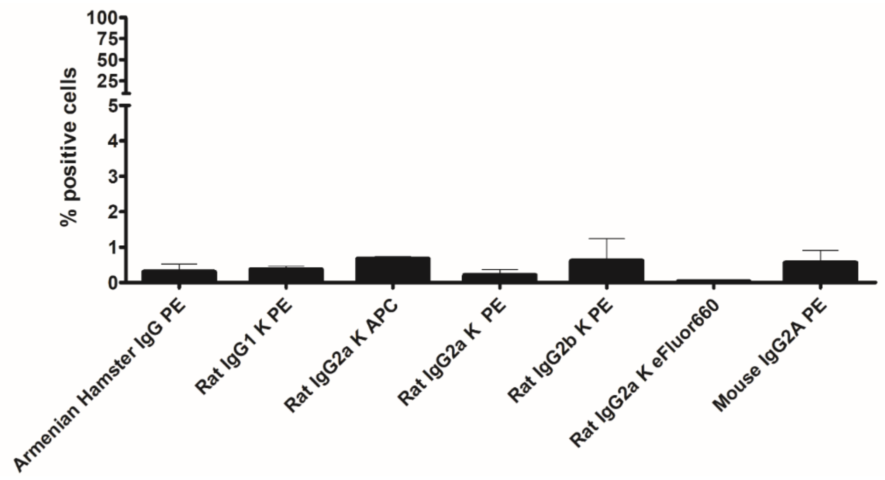

Figure S1 Measured quantity of positive cells [%] after labelling with the respective isotype control to the immunophenotyping of MSC by flow cytometry.

**Isotype control**	**Respective surface Marker**
Armenian Hamster IgG – PE	CD29
Rat IgG1 K – PE	CD73
Rat IgG2a K – APC	CD90, PDGFRα
Rat IgG2a K – PE	CD105, Sca-1
Rat IgG2b K – PE	CD44, CD45, Ter119
Rat IgG2a K – eFluor660	CD34
Mouse IgG2a - PE	Nestin

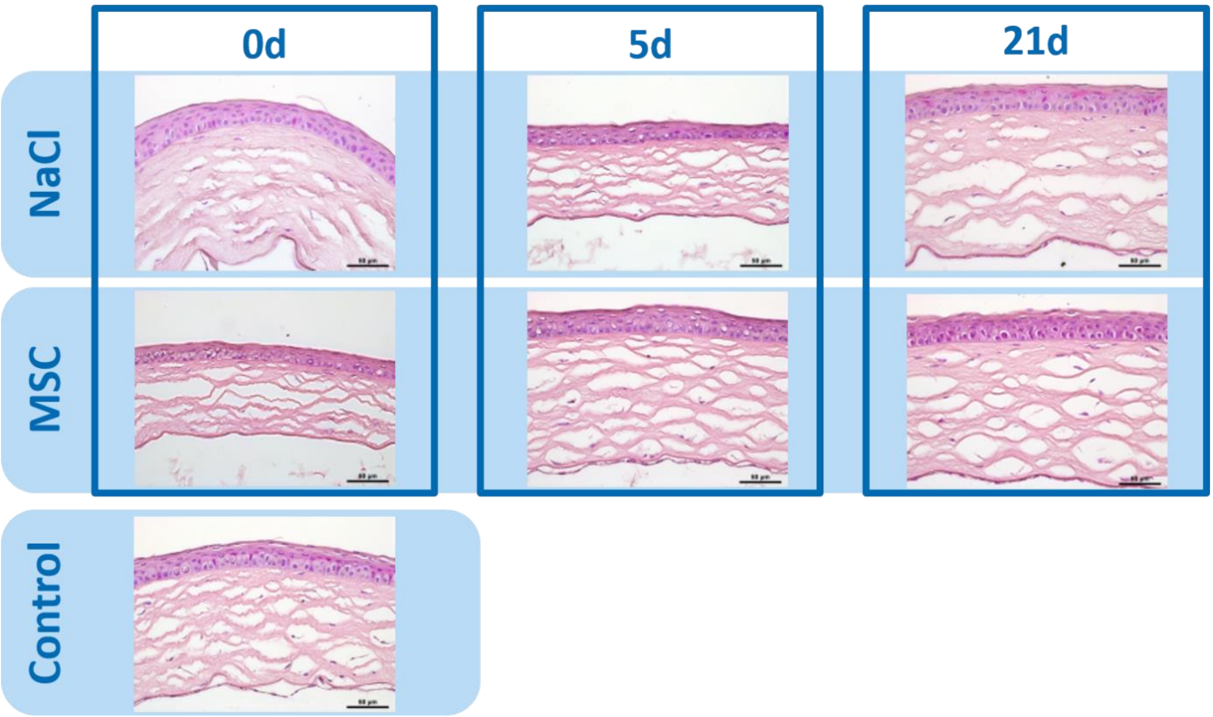

Figure S2 Hematoxylin and Eosin (HE) staining of corneal sections were stained and thickness of the corneal epithelium was measured at three defined measuring points using ImageJ. Scale bar: 50µm

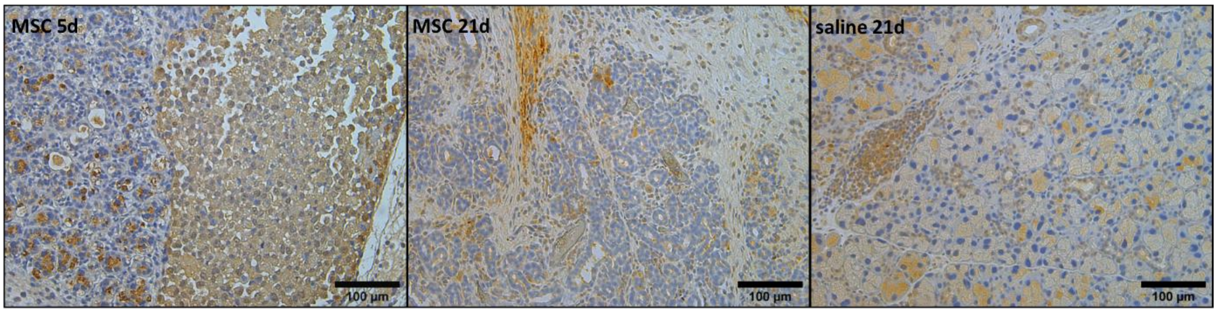

Figure S3 Immunohistochemical staining of GFP. Representative images showed that the transplanted MSC could be detected adjacent to the acinar structures within the connective tissue at 5 min, 5 days and 21 days after injection. Scale bar: 100µm

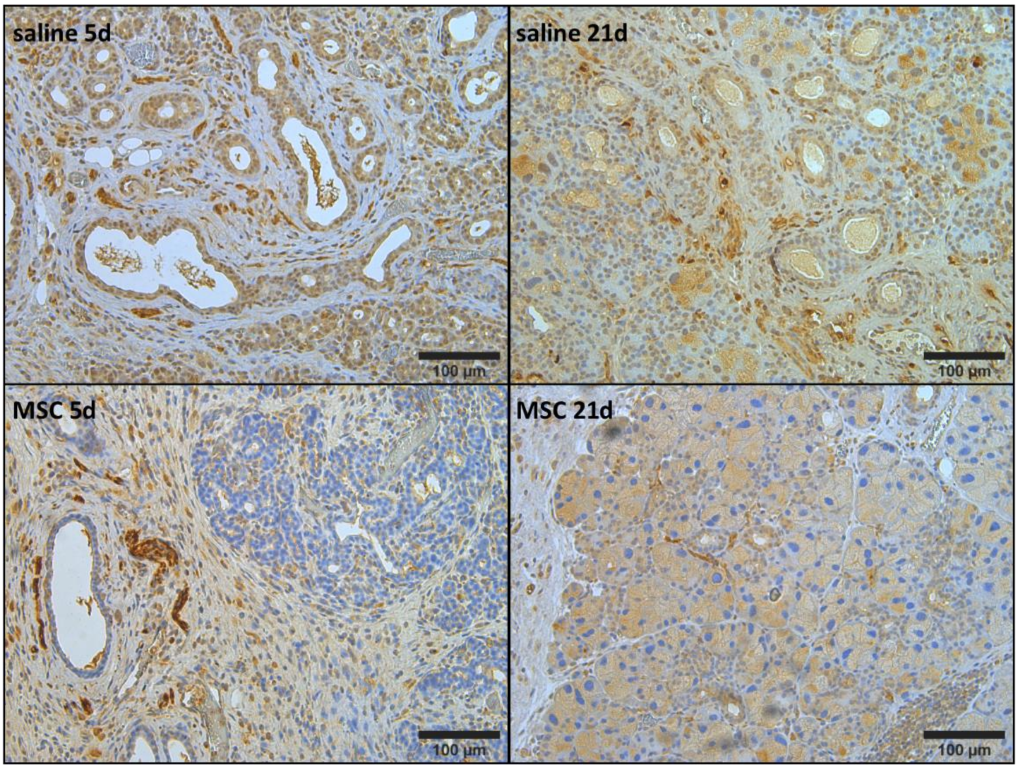

Figure S4 Immunohistochemical staining of Nestin. Representative images showed that nestin positive cells could be detected mainly in the stroma of (damaged) LG tissue. Nestin positive cells exhibited a spindle-shaped and elongated morphology. Scale bar: 100µm

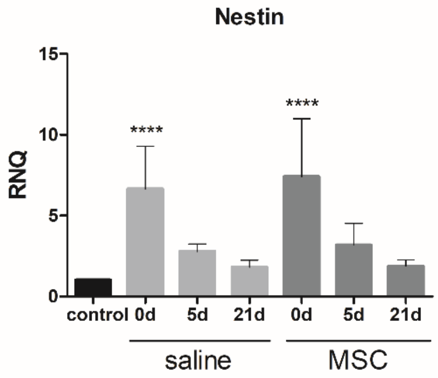

Figure S5 Gene expression analysis of Nestin. Nestin expression significantly increased after DL in both groups and then turned to control levels by day 21.

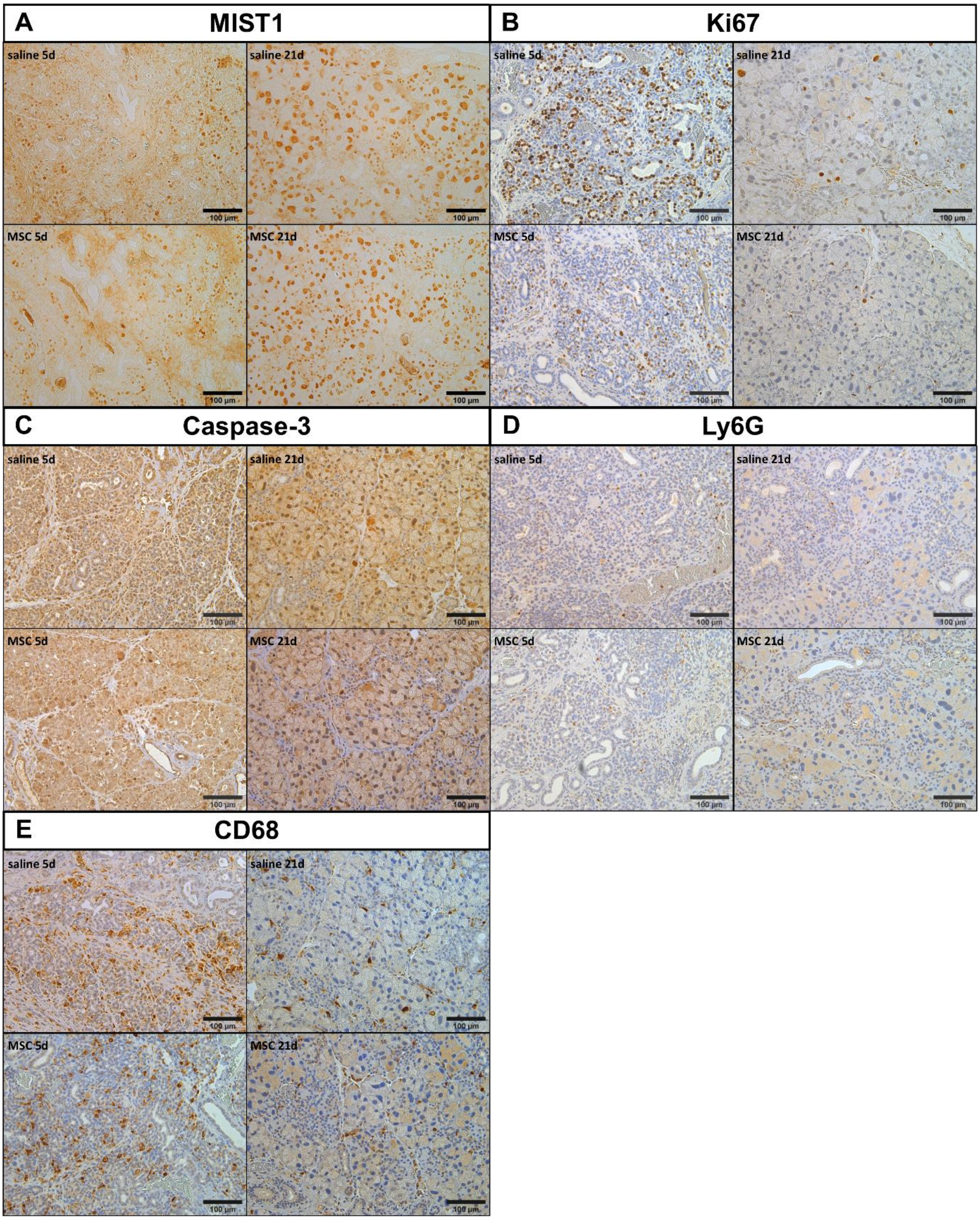

Figure S6 Overview of immunohistochemical staining. A: Representative images of MIST1 expression localize to the nucleus, therefore hematoxylin counterstain was omitted. A similar staining was detected after saline and MSC injection. B: Representative images of Ki67 display a higher number of Ki67 positive (proliferating) cells at day 5 after saline injection compared to MSC transplantation. C: Representative images of caspase-3 showed a darkish born staining of the cytoplasm of apoptotic cells. D: Representative images of Ly6G revealed a brown staining of small cells distributed throughout the whole lacrimal gland tissue. E: Representative images of CD68 detected positive cells distributed throughout the whole lacrimal gland tissue. Scale bar: 100µm

Supplementary Table S1: Primary Antibodies

Antibody	Conjugation	Introduction/f_{Verd}	#	Manufacturer	Application
CD16/CD32	-		553141	BD Bioscience,	Flowcytometry
CD29	PE	1µg/test	12-0291	eBioscience, San Diego, CA	Flowcytometry
CD34	eFluor660	1µg/test	50-0341	eBioscience, San Diego, CA	Flowcytometry
CD44	PE	0.125µg/test	12-0441	eBioscience, San Diego, CA	Flowcytometry
CD45	PE	0.03µg/test	12-0451	eBioscience, San Diego, CA	Flowcytometry
CD73	PE	0.125µg/test	12-0731	eBioscience, San Diego, CA	Flowcytometry
CD90.2	APC	0.06µg/test	17-0902	eBioscience, San Diego, CA	Flowcytometry
CD105	PE	0.5µg/test	12-1051	eBioscience, San Diego, CA	Flowcytometry
Nestin	PE	0.3µg/test	IC2736P	R&D Systems, Minneapolis, MN	Flowcytometry
PDGFRα	APC	1µg/test	17-1401	eBioscience, San Diego, CA	Flowcytometry
Sca-1	PE	0.125µg/test	12-5981	eBioscience, San Diego, CA	Flowcytometry
Ter119	PE	0.25µg/test	12-5921	eBioscience, San Diego, CA	Flowcytometry
Armenian Hamster IgG	PE	1µg/test	12-4888	eBioscience, San Diego, CA	Flowcytometry, Isotyp control
Rat IgG1 K	PE	0.125µg/test	12-4301	eBioscience, San Diego, CA	Flowcytometry, Isotyp control
Rat IgG2a K	APC	1µg/test	17-4321	eBioscience, San Diego, CA	Flowcytometry, Isotyp control
Rat IgG2a K	PE	0.5µg/test	12-4321	eBioscience, San Diego, CA	Flowcytometry, Isotyp control
Rat IgG2b K	PE	0.25µg/test	12-4031	eBioscience, San Diego, CA	Flowcytometry, Isotyp control
Rat IgG2a K	eFluor660	1µg/test	50-4321	eBioscience, San Diego, CA	Flowcytometry, Isotyp control
Mouse IgG2a	PE	0.3µg/test	IC003P	R&D Systems, Minneapolis, MN	Flowcytometry, Isotyp control
Nestin	-	500	839801	BioLegend, San Diego, CA	Immunohistochemistry, paraffin
GFP	-	700	ab13970	Abcam, Cambridge, UK	Immunohistochemistry, paraffin
Ki67	-	100	ab16667	Abcam, Cambridge, UK	Immunohistochemistry, paraffin
Caspase-3	-	100	ab4051	Abcam, Cambridge, UK	Immunohistochemistry, paraffin
CD68	-	300	ab125212	Abcam, Cambridge, UK	Immunohistochemistry, paraffin

Ly6G	-	100	MAB1037	R&D Systems, Minneapolis, MN	Immunohistochemistry, paraffin
MIST1	-	50	sc-80983	Santa Cruz, Dallas, Tx	Immunohistochemistry, paraffin
Affini Pure Fab Fragment Donkey Anti-Mouse IgG (H+L)		40	715-007-003	Jackson ImmunoResearch Laboratories, West Grove, PA	Immunohistochemistry, paraffin
Donkey Anti-Chicken IgY	Biotin	300	703-065-155	Jackson ImmunoResearch Laboratories, West Grove, PA	Immunohistochemistry, paraffin
Horse Anti-Mouse IgG (H+L)	Biotin	300	BA-2000	Vector Laboratories, Burlingame, CA	Immunohistochemistry, paraffin
Horse Anti-Rabbit IgG (H+L)	Biotin	300	BA-1100	Vector Laboratories, Burlingame, CA	Immunohistochemistry, paraffin
Goat Anti-Rat IgG	Biotin	100	BA-9401	Vector Laboratories, Burlingame, CA	Immunohistochemistry, paraffin
Nestin	-	5000	NB-100-1604	Novus Biologicals, Centennial, CO	Western Blot
STAT1	-	1000	9172	Cell Siganling, Denvers, MA	Western Blot
GFP	-	5000	ab13970	Abcam, Cambridge, UK	Western Blot
Lipocalin-2	-	800	ab216462	Abcam, Cambridge, UK	Western Blot
IL-6	-	500	MAB406	R&D Systems, Minneapolis, MN	Western Blot
TNFα	-	500	AF-410	R&D Systems, Minneapolis, MN	Western Blot
Goat Anti-Mouse IgG	HRP	10000	A3682	Sigma-Aldrich, St. Louis, MO	Western Blot
Goat Anti-Rabbit IgG	HRP	20000	A9169	Sigma-Aldrich, St. Louis, MO	Western Blot
Donkey Anti-Chicken IgY	HRP	10000	AP194P	Sigma-Aldrich, St. Louis, MO	Western Blot
Donkey Anti-Rat IgG	HRP	10000	AP183P	Sigma-Aldrich, St. Louis, MO	Western Blot
Rabbit Anti-Goat IgG	HRP	3000	81-1620	Invitrogen/Thermo Fisher Waltham, MS	Western Blot
Nestin	-	50	sc-33677	Santa Cruz, Dallas, Tx	Immunocytochemistry
Alexa Fluor 594 AffiniPure Donkey Anti-Mouse IgG		400	715-585-150	Jackson Immunoresearch	Immunocytochemistry

Supplementary Table S2: Primer sequences for real-time PCR analysis

Primer	mRNA	Sequenz
mTBP-F127	TATA-box binding protein	GCA GTG CCC AGC ATC ACT AT
mTBP-R268		AAG GCT GTT GTT CTG GTC CAT
RPS6-F	Ribosomal protein s6	CTT TTT CGT GAC GCC TCC CA
RPS6-R		GGG AAG GAG ATG TTC AGC TTC A
mOpn-F31	Osteopontin	CCT TGC TTG GGT TTG CAG TC
mOpn-R153		TGG TCG TAG TTA GTC CCT CAG A
FABP4-F	Fatty acid binding protein	TGA AAT CAC CGC AGA CGA CA
FABP4-R		ACA CAT TCC ACC ACC AGC TT
mIL-6-F569	Interleukin-6	GTG GCT AAG GAC CAA GAC CA
mIL-6-R663		TAA CGC ACT AGG TTT GCC GA
mLcn2-F356	Lipocalin-2	GGC CAG TTC ACT CTG GGA AA
mLcn2-R446		TGG CGA ACT GGT TGT AGT CC
mSTAT1-F79	Signal transducer and activator of transcription 1	CCT GTC ATC CCG CAG AGA GA
mSTAT1-R158		CGG CAG GAT AAG AGA GCC AA
mMIST1-F60	Basic Helix-Loop-Helix Family, Member A15	CCT CGA ATC CCC AGT TGG AA
mMIST1-R176		CTG TGT CCT GCA TGG GTG TT
mRbmy-F1057	RNA-binding motif (RRM) gene on Y chromosome	GAT TCC ATG AGG CAC CAT CT
mRbmy-R1149		ATG GTT CTC CTC TTC CAC CA
mTnfa-F318	Tumor necrosis factor alpha	CTG AAC TTC GGG GTG ATC GG
mTnfa-R415		TGG TTT GTG AGT GTG AGG GT
mNestin-F	Nestin	GCA GGC CAC TGA AAA GTT CC
mNestin-R		GCA GAG TCC TGT ATG TAG CCA

Supplementary Table S3: Loading for Western Blot Analysis

Gel 1	treatment	Time point
1	control	
2	DL+saline injection	0d
3	DL+saline injection	0d
4	DL+saline injection	5d
5	DL+saline injection	5d
6	DL+saline injection	21d
7	DL+saline injection	21d
8	DL+MSC injection	0d
9	DL+MSC injection	0d
10	DL+MSC injection	5d
11	DL+MSC injection	5d
12	DL+MSC injection	21d

Gel 2		
13	control	
14	control	
15	DL+saline injection	0d
16	DL+saline injection	0d
17	DL+saline injection	5d
18	DL+saline injection	5d
19	DL+saline injection	21d
20	DL+MSC injection	0d
21	DL+MSC injection	0d
22	DL+MSC injection	5d
23	DL+MSC injection	21d
24	DL+MSC injection	21d

Gel 3		
25	control	
26	control	
27	DL+saline injection	0d
28	DL+saline injection	5d
29	DL+saline injection	21d
30	DL+saline injection	21d
31	DL+MSC injection	0d
32	DL+MSC injection	5d
33	DL+MSC injection	5d
34	DL+MSC injection	21d
35	DL+MSC injection	21d

Gel 4

36	control	
37	DL+saline injection	0d
38	DL+saline injection	5d
39	DL+saline injection	21d
40	DL+MSC injection	0d
41	DL+MSC injection	5d
42	DL+MSC injection	21d

All Blots were performed in parallel. Representative figures are provided in the manuscript.

Abbreviations:

DL – duct ligation

MSC – mesenchymal stem cells

M - Marker

TNFα

M	1	2	3	4	5	6	7	8	9	10	11	12	M	TNF a

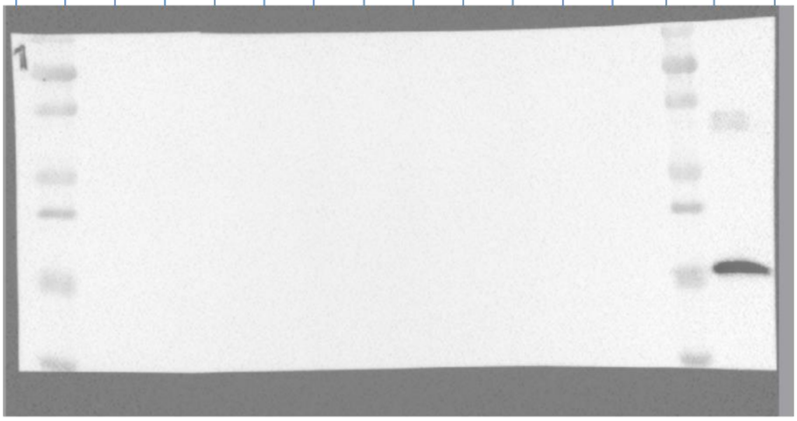

TNFα dimer 34kDa

TNFα monomer 17kDa

M	13	14	15	16	17	18	19	20	21	22	23	24	M	TNFa

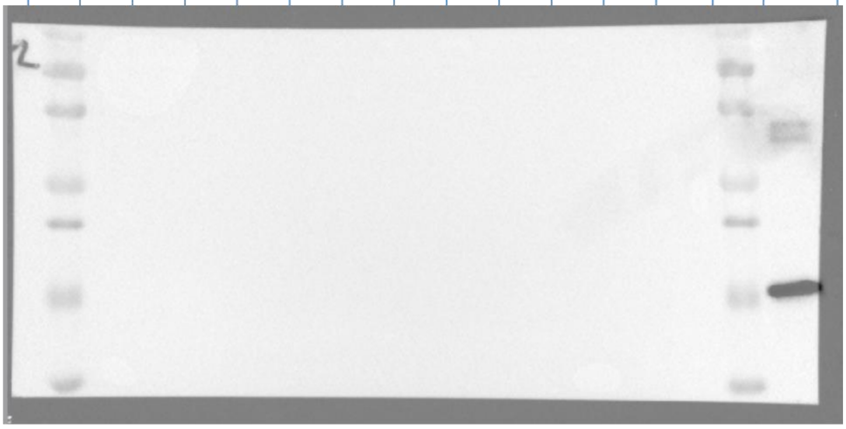

M	25	26	27	28	29	30	31	32	33	34	35	M	TNFa

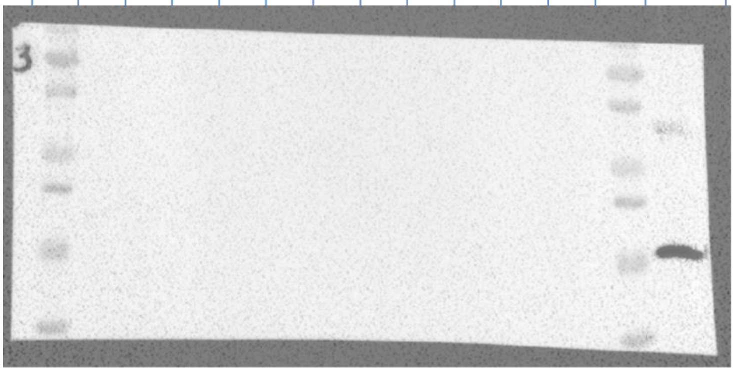

M	36	37	38	39	40	41	42	M	TNFa

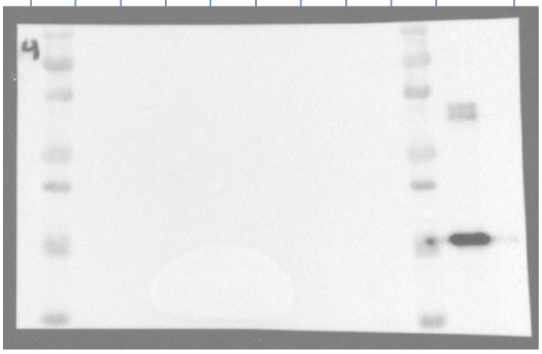

Lipocalin-2

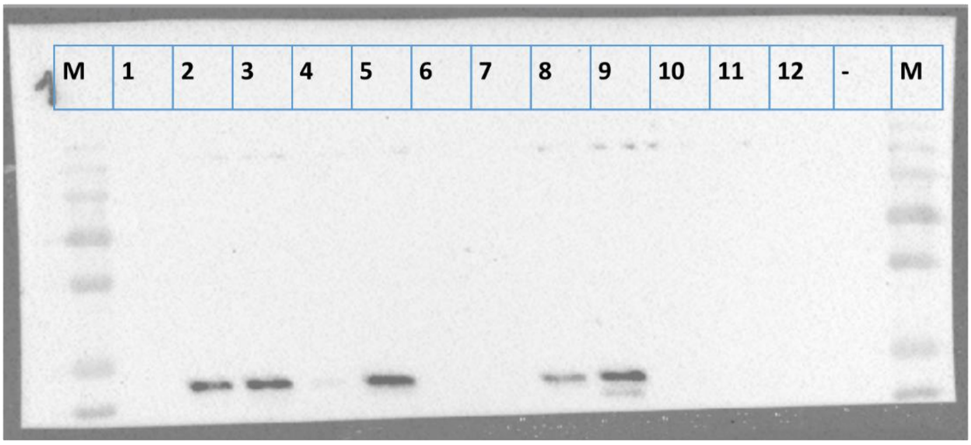
M
1
2
3
4
5
6
7
8
9
10
11
12
-
M

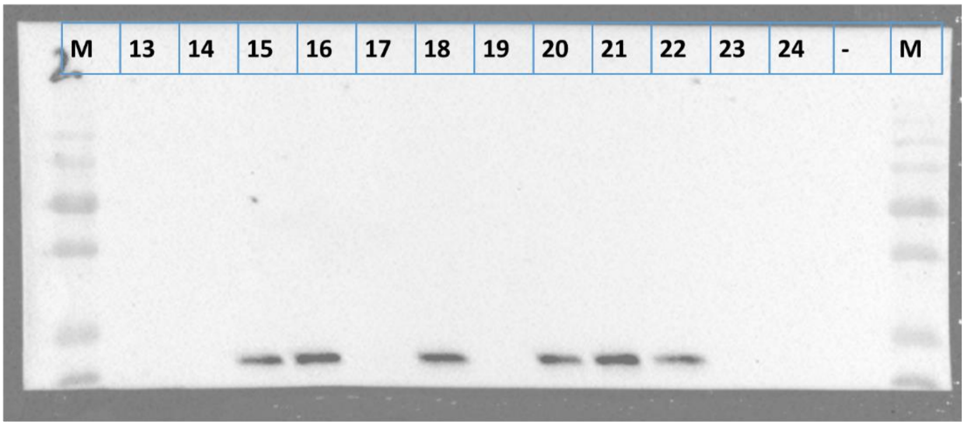
M
13
14
15
16
17
18
19
20
21
22
23
24
-
M

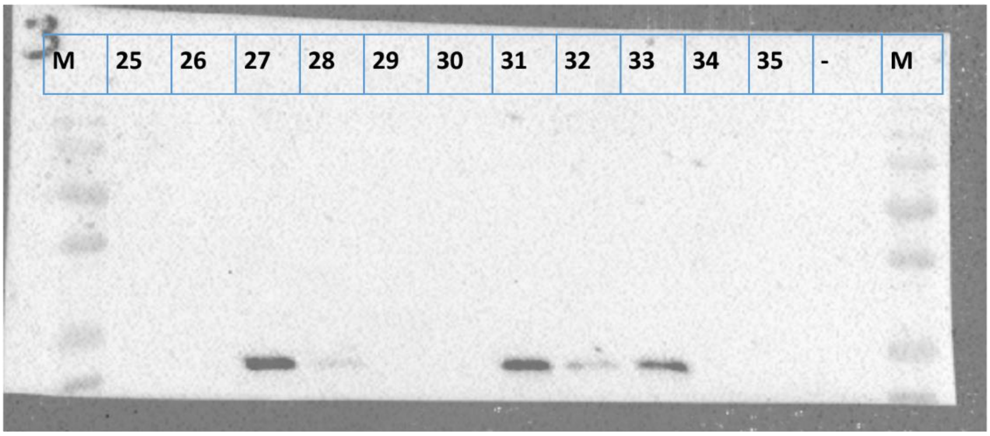
M
25
26
27
28
29
30
31
32
33
34
35
-
M

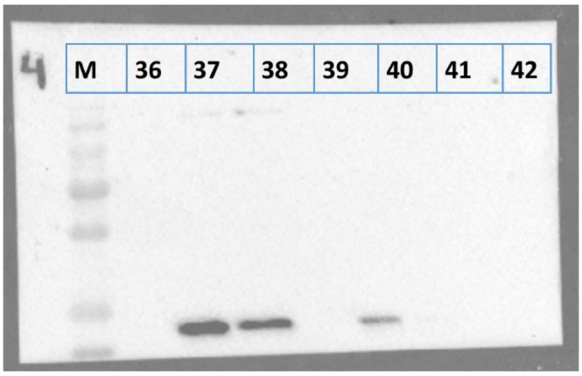
M
36
37
38
39
40
41
42

STAT1 and GFP

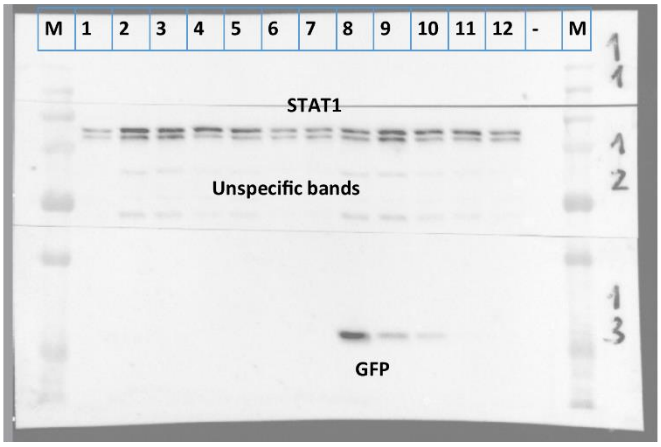

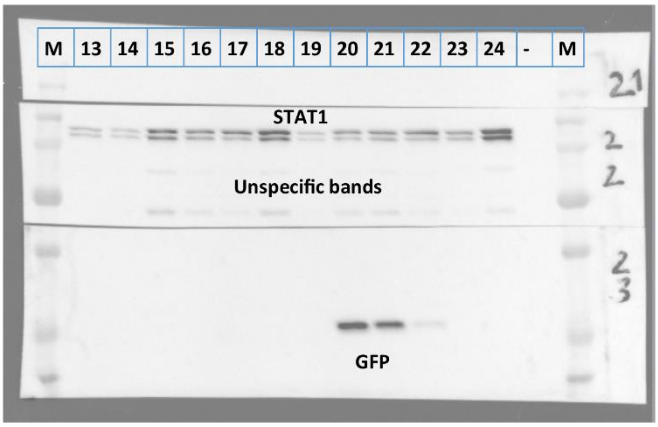

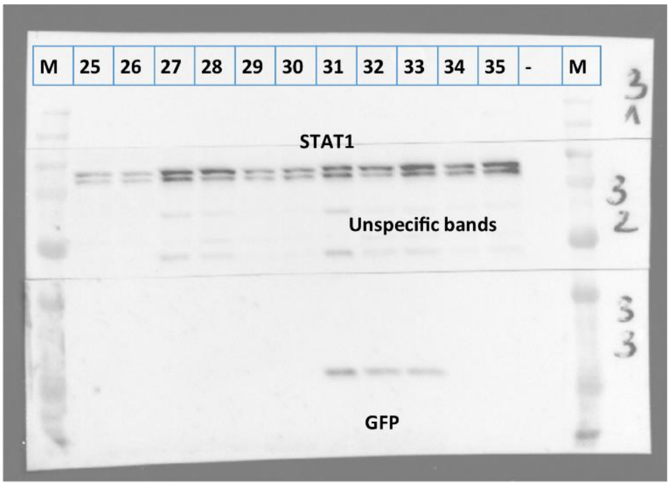

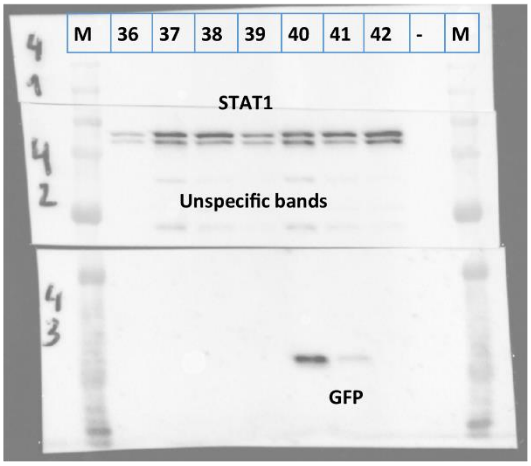

Nestin

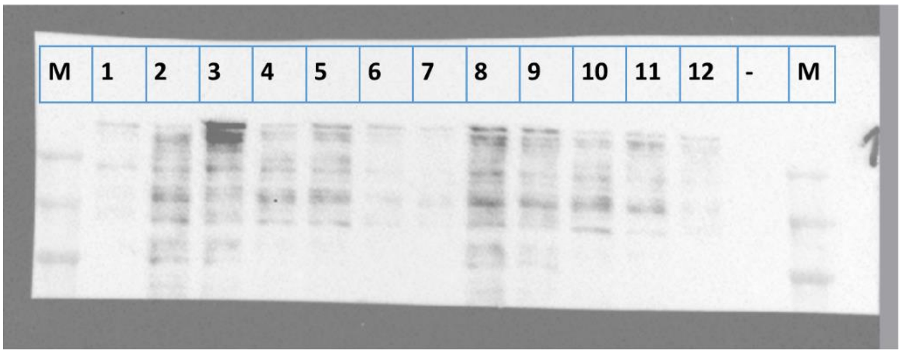

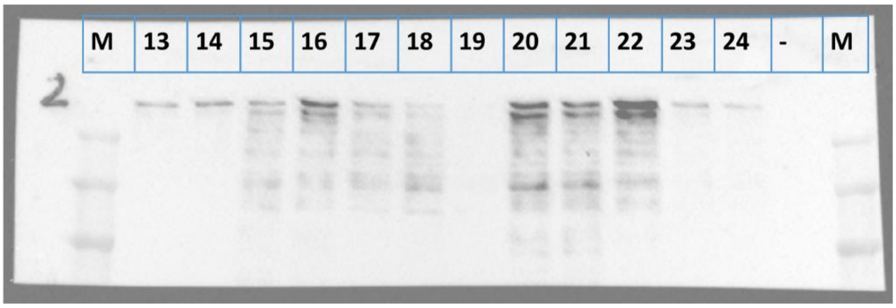

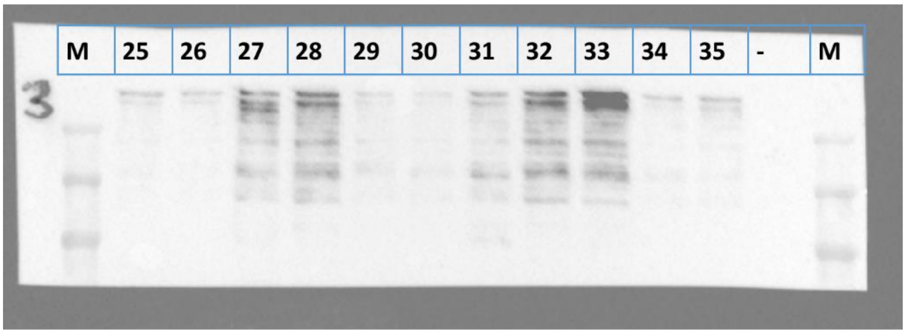

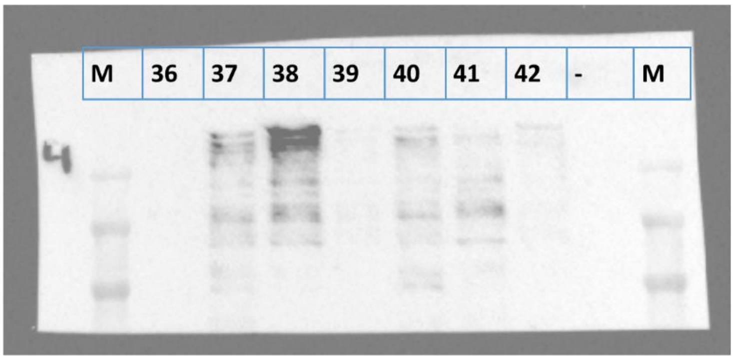

Molecular weight of nestin is highly variable due to posttranslational modifications and range from 140-250kDa [1]. Therefore, bands in the range of this size were analzed.

1. Hadavi *et al.* „Production of monoclonal antibody against human nestin“, Avicenna J Med Biotechnol. 2010 Apr-Jun; 2(2):69-77

IL-6

M	1	2	3	4	5	6	7	8	9	10	11	12	IL-	M

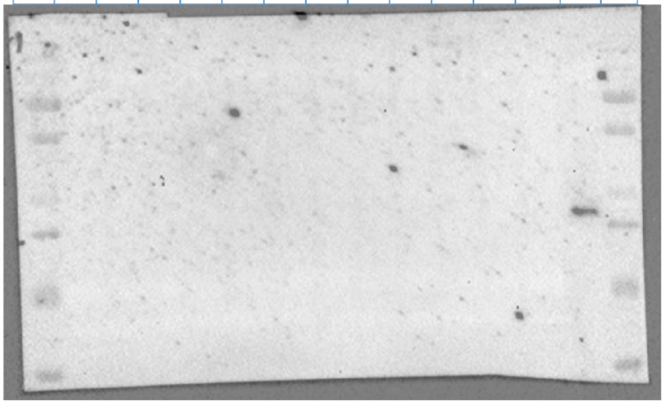

M	13	14	15	16	17	18	19	20	21	22	23	24	IL-6	M

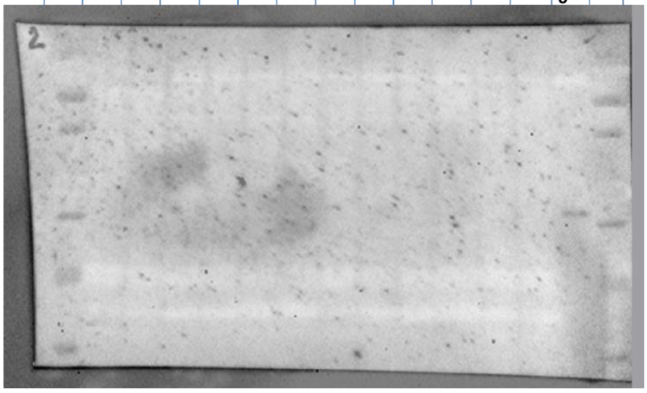

M	25	26	27	28	29	30	31	32	33	34	35	IL-6	M

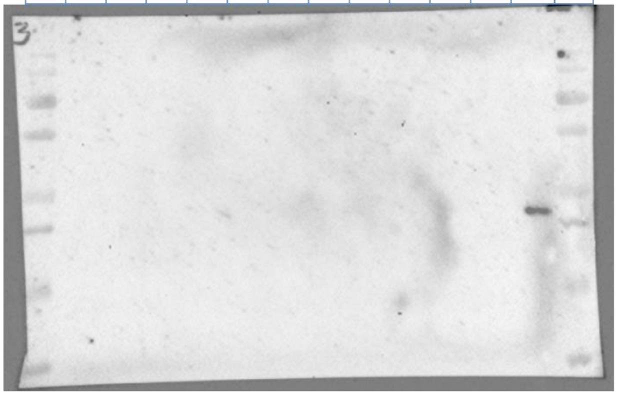

M	36	37	38	39	40	41	42	IL-6

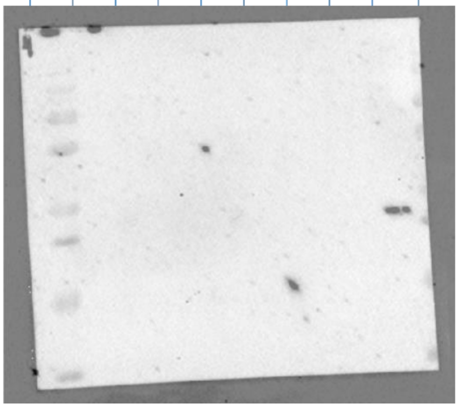

Supplement: Supplementary file 1 — Supplemental Material [file 41598_2019_54840_MOESM1_ESM.pdf]
